# A Brain‐Penetrant Nanobody Reveals GSK3β‐Driven Proline‐Directed Phosphorylation as a Master Regulator of Ischemic Neurodegeneration

**DOI:** 10.1002/advs.76004

**Published:** 2026-06-09

**Authors:** Lan Li, Muyang Li, Lei Sun, Ying Yang, Yuanshun Wu, Ziyi Yin, Anni Wang, Peiyang Zhou, Shaoxiang Luo, Jian Chen, Jun Qin, Zhibing Ai, Zilong Yuan, Zhiqiang Dong, Min Zhang

**Affiliations:** ^1^ College of Biomedicine and Health College of Life science and Technology Huazhong Agricultural University Wuhan China; ^2^ Hubei Clinical Research Center of Central Nervous System Repair and Functional Reconstruction Taihe Hospital Hubei University of Medicine Shiyan Hubei China; ^3^ Department of Neurology Xiangyang No.1 People's Hospital Hubei University of Medicine Xiangyang Hubei China; ^4^ School of Traditional Chinese Medicine Beijing University of Chinese Medicine Beijing China; ^5^ Wuhan Proteinligand Biotechnology Co., Ltd Wuhan China; ^6^ Hubei Cancer Hospital Tongji Medical College Wuhan China

**Keywords:** GSK3β, HFn nanoparticle, ischemia stroke, nanobody, neuroprotection, proline‐directed phosphorylation

## Abstract

Serine/threonine‐proline (S/T‐P) phosphorylation is a fundamental mechanism maintaining cellular homeostasis. Although glycogen synthase kinase 3β (GSK3β) is a key regulator in ischemic stroke, the contribution of its proline‐directed kinase activity to cellular dysfunction and disease progression remains unclear. Here, we developed Nb.29E9, a nanobody that selectively targets the proline‐directed kinase domain of GSK3β. Under ischemic conditions, Nb.29E9 inhibited S/T‐P phosphorylation of key substrates, including RNA‐binding motif protein 38 (RBM38), HIF1α, and p53, thereby enhancing neuronal and microglial viability while reducing oxidative stress and neuroinflammation. Phosphoproteomic analysis revealed broad reprogramming of S/T‐P phosphorylation networks. In mice after ischemic injury, Nb.29E9 delivered via a brain‐penetrant, MMP‐9‐responsive nanoparticle reduced infarct volume, restored neurovascular integrity, and improved motor function. Mechanistically, Nb.29E9 corrected pathological hyperphosphorylation of SMAD2/3‐Thr8 (TGFβ signaling), calcium/calmodulin‐dependent protein kinase kinase 2 (CAMKK2)‐Ser495 (AMPK pathway), and AKT1 substrate 1 (AKT1S1)‐Ser183 (mTORC1 regulation). These findings demonstrate that GSK3β’s intrinsic proline‐directed kinase activity drives ischemic neurodegeneration, establishing its pathogenic role in vivo.

## Graphical abstract

A brain‐targeted nanoparticle enables delivery of a therapeutic nanobody (Nb.29E9) that inhibits pathogenic GSK3β signaling. This intervention restores AMPK/mTORC1/TGFβ homeostasis, attenuates neuroinflammation and oxidative stress, and promotes long‐term functional recovery after ischemic stroke.

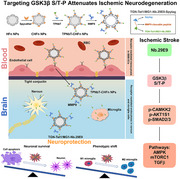

## 1 Introduction

Stroke remains a leading cause of mortality and long‐term disability worldwide, with ischemic stroke accounting for approximately 80% of all cases [1]. Recent advances have highlighted the central role of post‐translational modifications, particularly protein phosphorylation, in mediating ischemic neuronal death and survival pathways [2, 3]. Upon cerebral hypoperfusion, the ischemic penumbra gradually evolves into the irreversible ischemic core [4, 5]. Cessation of cerebral blood flow rapidly depletes oxygen and glucose, triggering energy failure, ion dyshomeostasis, and acidosis [6]. These events initiate pathological cascades including excitotoxicity and oxidative stress in the acute phase, followed by inflammation and apoptosis in the subacute phase [2]. The molecular regulation of these cascades involves dynamic post‐translational modifications, with protein phosphorylation playing a central role [7, 8]. Among phosphorylation regulators, GSK3β is a pivotal kinase, highly expressed in neurons and glia, that regulates neuronal survival, neuroinflammation, and synaptic plasticity [9, 10, 11]. In ischemic stroke, aberrant GSK3β activation exacerbates oxidative stress, neuroinflammation, and apoptosis, accelerating ischemic core expansion [12, 13]. However, the precise spatiotemporal dynamics of pathogenic phosphorylation events mediated by GSK3β, particularly its S/T‐P kinase activity, remain uncharacterized, limiting targeted therapeutic development [14, 15]. While pharmacological GSK3β inhibition shows neuroprotective effects, current strategies cannot distinguish disease‐driving phosphorylation from physiological signaling and face bioavailability limitations [16, 17].

GSK3β possesses two mechanistically distinct kinase activities: primed‐substrate‐dependent phosphorylation and S/T‐P phosphorylation [18]. Its catalytic output is primarily regulated by two antagonistic phosphorylation events. Autophosphorylation at Tyr216 promotes both activities by stabilizing the active kinase conformation, whereas Ser9 phosphorylation preferentially suppresses primed‐substrate recognition by sterically blocking the phosphate‐binding pocket, thereby sparing S/T‐P activity [19]. In addition to phosphorylation, protein‐protein interactions (PPIs) exert critical control over GSK3β function. For example, the Axin scaffolding complex spatially positions GSK3β to facilitate β‐catenin phosphorylation [20]. Increasing evidence highlights that proline‐directed phosphorylation, although traditionally considered secondary to primed‐substrate regulation, represents a critical yet incompletely characterized pathway in central nervous system disorders [21, 22]. This activity exhibits substrate‐specific and context‐dependent dysregulation in neurodegenerative diseases, potentially altering protein conformation, stability, and aggregation propensity [23, 24]. However, the upstream and structural determinants governing its spatiotemporal dynamics, including the influence of priming kinases, scaffold interactions, and secondary post‐translational modifications, remain insufficiently defined [25, 26].

Functionally, selective modulation of GSK3β’s two activities produces distinct outcomes in neurites [27]. Inhibition of primed‐substrate phosphorylation, as observed for CRMP2 or APC, enhances microtubule stability and promotes axon elongation, whereas maintenance of S/T‐P phosphorylation, such as MAP1B modification, supports microtubule dynamics required for growth‐cone motility and branching [28, 29, 30]. These observations suggest that precision targeting of the S/T‐P phosphorylation module, rather than non‐selective GSK3β inhibition, offers a mechanistically rational strategy to separate pathological phosphorylation from physiological signaling [27]. Such an approach could preserve essential primed‐substrate signaling for neuronal polarity while preventing disease‐associated dysregulation [9].

Our previous work identified a core regulatory mechanism governing GSK3β’s S/T‐P kinase activity. We found that eukaryotic translation initiation factor 4E type 2 (eIF4E2) interacts with GSK3β through a conserved binding motif and thereby selectively sustains its S/T‐P phosphorylation function [14]. This eIF4E2‐GSK3β module maintains phosphorylation at S/T‐P sites on diverse substrates, including RBM38 (Ser195), HIF1α (Ser589), and multiple p53 (Ser315) sites [14, 15]. Hypoxic stress disrupts this regulatory module through GSK3β S‐nitrosylation, a modification that operates independently of the canonical PI3K/AKT‐Ser9 inhibitory pathway [14]. These findings define a noncanonical, stress‐responsive mechanism for tuning GSK3β substrate selectivity.

Concurrently, drug discovery efforts have focused on three complementary structural strategies to achieve precise pharmacological modulation of GSK3β. The first involves refinement of ATP‐competitive inhibitors through hinge‐binding motif optimization and exploitation of adjacent subpockets to enhance potency, selectivity, and central nervous system penetration [31, 32]. The second encompasses non‐ATP‐based approaches, including substrate‐competitive peptides and small molecules that engage primed‐substrate docking grooves or target validated allosteric sites outside the highly conserved ATP‐binding domain [33]. The third strategy entails the development of dual PI3K/AKT‐GSK3β inhibitors designed to attenuate compensatory signaling pathways that limit the efficacy of single‐target agents [34]. Despite these advances, most existing compounds still lack stringent isoform specificity (GSK3α vs. GSK3β) and activity‐form selectivity (discrimination between primed‐substrate and proline‐directed kinase functions), which remains a major barrier to therapeutic precision [35].

Nanobodies (Nbs, ∼15 kDa, ∼2–3 nm) offer distinct structural advantages for addressing these limitations [36]. Their elongated complementarity‐determining region 3 (CDR3) loops enable access to shallow or conformationally dynamic surface clefts that are often inaccessible to conventional small molecules, permitting highly specific blockade of protein‐protein interfaces [37]. By binding with high spatial precision to regulatory interfaces, a nanobody can sterically occlude critical interactions and, in some cases, induce conformational perturbations that alter active‐site geometry or regulatory loop mobility, thereby achieving functional modulation beyond simple orthosteric inhibition [38]. Based on our structural characterization of the eIF4E2‐GSK3β regulatory interface, we hypothesized that a targeted nanobody might achieve three levels of selectivity: (1) GSK3β isoform specificity, (2) proline‐directed activity preference, and (3) stress‐state dependence. This approach would allow both mechanistic investigation of ischemic stroke pathways and development of precision therapeutics.

Here, we report the development of Nb.29E9, a conformation‐specific nanobody that targets the GSK3β domain required for proline‐directed phosphorylation. Nb.29E9 selectively inhibited S/T‐P phosphorylation and conferred robust neuroprotection in oxygen glucose deprivation/reperfusion (OGD/R) models by attenuating oxidative stress and neuroinflammation. To achieve efficient central nervous system (CNS) delivery, we incorporated Nb.29E9 into a multifunctional ferritin‐based platform (TPNbT‐CHFn), which combines blood‐brain barrier (BBB) translocation, neuron/microglia targeting, and microenvironment‐responsive release. In middle cerebral artery occlusion/reperfusion (MCAO/R) animals, nanoparticle‐delivered Nb.29E9 significantly reduced infarct volume, preserved neurovascular integrity and improved motor recovery by concurrently mitigating apoptosis, oxidative stress and inflammatory responses. These findings suggest that proline‐directed GSK3β activity may function as a pathogenic driver in ischemic injury, and that activity‐specific kinase modulation via nanobody technology warrants evaluation as a selective therapeutic strategy.

## 2 Results

### 2.1 A Conformation‐Specific Nanobody, Nb.29E9, Selectively Binds and Inhibits GSK3β’s Proline‐Directed Kinase Activity

**FIGURE 1:**
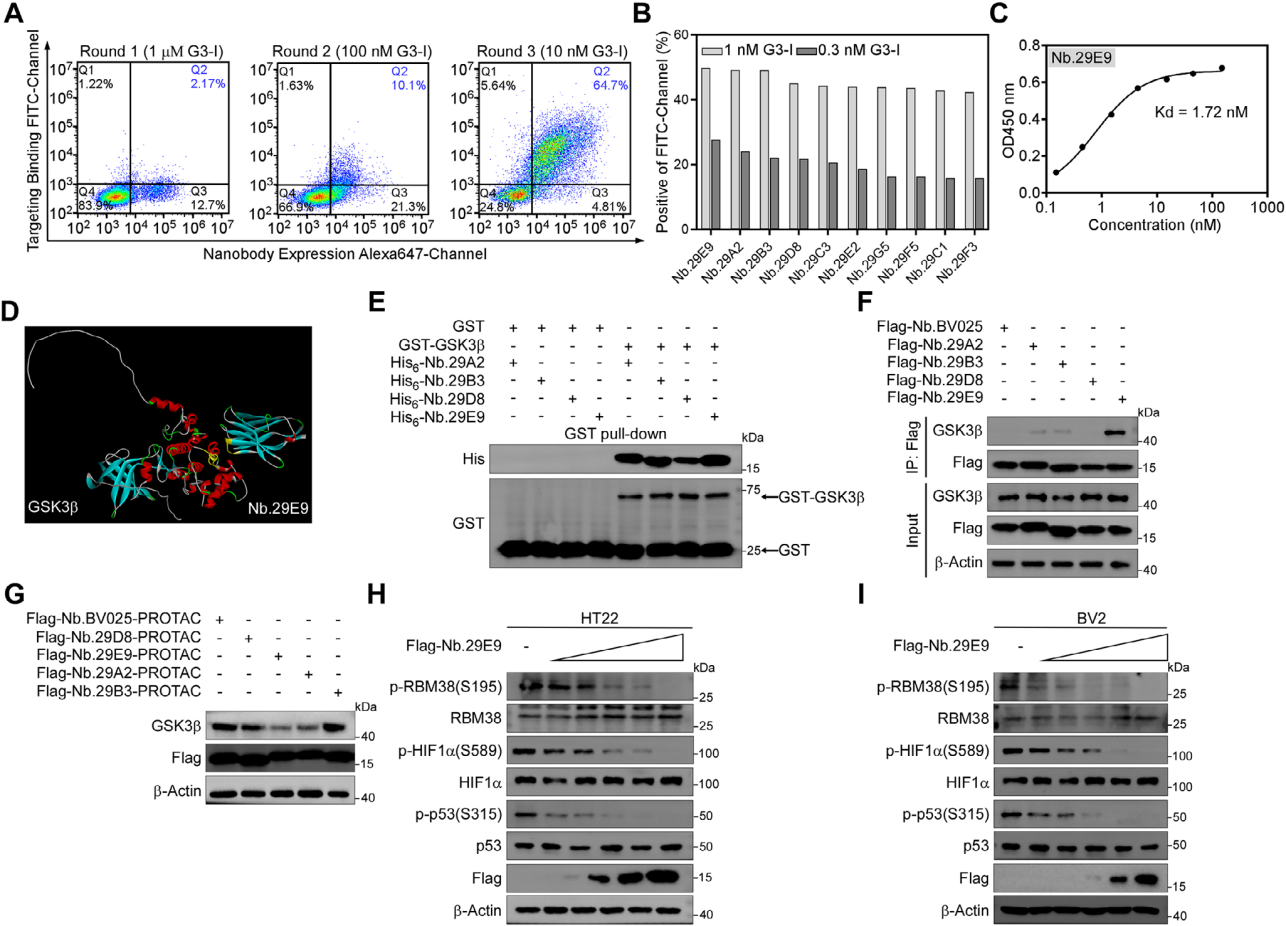
Screening and validation of GSK3β‐targeting nanobodies. (A) FACS‐based enrichment of yeast‐displayed clones using synthetic peptide G3‐I. (B) Flow cytometry of selected clones at different antigen concentrations. (C) ELISA validation of Nb.29E9 binding to G3‐I (*n* = 3 per group). (D) Docking model showing Nb.29E9 engagement of the eIF4E2‐GSK3β interface. (E) GST pull‐down confirming in vitro binding of candidate nanobodies to recombinant GSK3β. (F) Co‐IP analysis of Nb.29E9 binding to endogenous GSK3β. (G) Nb.29E9‐PROTAC induces SPOP‐dependent degradation of GSK3β under normoxia and hypoxia. (H, I) Western blots in HT22 and BV2 cells showing Nb.29E9 inhibition of RBM38 (pS195), HIF1α (pS589), and p53 (pS315) phosphorylation in a dose‐dependent manner. See also Figure S1.

To elucidate the regulatory mechanism of GSK3β’s proline‐directed kinase activity, we developed GSK3β‐targeting nanobodies using a synthetic peptide antigen (G3‐I, sequence CSRLLEYPTARL) derived from a GSK3β surface loop (residues 318–329 of GSK3β) that partially mediates the eIF4E2‐GSK3β interaction. Employing our established protocol, we constructed a fully synthetic yeast‐displayed nanobody library and conducted three rounds of fluorescence‐activated cell sorting (FACS). This progressive enrichment strategy significantly increased the double‐positive clone population from 2.17% to 64.7%, with distinct subpopulation segregation indicating effective selection of high‐affinity nanobodies (Figure 1A). From the final FACS output, single‐clone cultures were established and binding affinity was validated by flow cytometry, identifying ten high‐affinity candidate clones (Figure 1B). Further ELISA validation using the antigen (G3‐I) confirmed strong binding affinity of the high‐affinity candidate clone Nb.29E9 (Figure 1C). CDR3 conservation analysis was performed by next‐generation sequencing (NGS) coupled with WebLogo 3.7. (Figure S1A). Molecular docking simulations using HADDOCK revealed that Nb.29E9, the highest‐affinity clone, specifically engages the eIF4E2‐GSK3β interaction interface via its CDR3 loop (Figure 1D).

Among these ten clones, four (including Nb.29E9) with the highest ELISA signals were selected for further biochemical characterization. GST pull‐down assays demonstrated specific binding of all four candidate nanobodies to recombinant GSK3β, whereas GST alone did not precipitate the kinase (Figure 1E). In co‐immunoprecipitation (Co‐IP) analysis of endogenous GSK3β, Nb.29E9 showed markedly stronger binding compared with the other clones tested (Figure 1F). To verify cellular target engagement, we utilized a nanobody‐based proteolysis‐targeting chimera (Nb‐PROTAC) composed of a chimeric nanobody‐SPOP (speckle‐type POZ protein) fusion. Nb.29E9 effectively mediated SPOP‐dependent degradation of endogenous GSK3β (Figure 1G), with comparable efficiency under normoxic and hypoxic conditions (Figure S1B).

We next evaluated the functional consequences of Nb.29E9 binding on GSK3β’s catalytic activity, using canonical eIF4E2‐GSK3β pathway substrates as phospho‐markers: RBM38 (pS195), HIF1α (pS589), and p53 (pS315) [14]. Western blot analysis demonstrated that Nb.29E9 dose‐dependently inhibited GSK3β’s Ser/Thr‐Pro (S/T‐P) kinase activity (Figure 1H,I). Notably, overexpression of Nb.29E9 under basal (non‐ischemic) conditions modestly reduced the viability of HT22 neurons and BV2 microglia (Figure S1C), likely reflecting a requirement for tonic S/T‐P signaling. This observation highlights the importance of stress‐state‐dependent delivery, motivating the design of our brain‐targeting nanoplatform. Collectively, these structural and functional data demonstrate that Nb.29E9 precisely targets the eIF4E2‐GSK3β interaction interface and functions as a selective intracellular inhibitor of GSK3β’s proline‐directed kinase activity.

### 2.2 Nb.29E9 Confers Neuroprotection in OGD/R by Attenuating Apoptosis, Oxidative Stress, and Neuroinflammation

**FIGURE 2:**
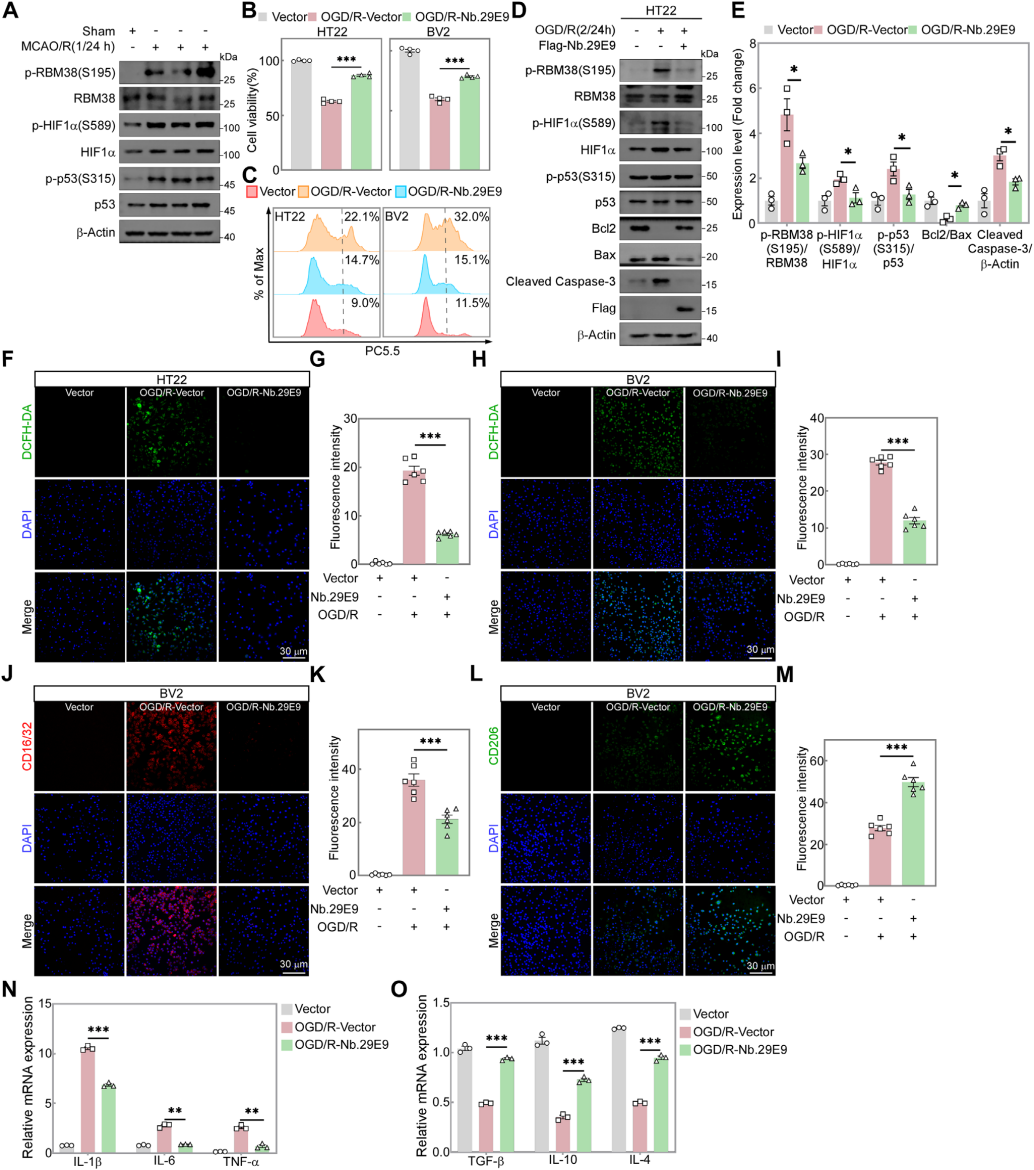
Nb.29E9 confers neuroprotection under OGD/R. (A) Western blot analysis of the ipsilateral cortex showing MCAO/R‐induced phosphorylation of canonical GSK3β substrates (p‐RBM38 S195, p‐HIF1α S589, p‐p53 S315). (B) Viability of HT22 and BV2 cells following OGD/R. Cells were transfected with empty vector (Vector) or Nb.29E9 prior to OGD/R induction. Data are mean ± SEM (*n* = 4 per group). ^***^ *p* < 0.001 vs. the indicated comparison group; one‐way ANOVA with Tukey's multiple comparisons test. (C) Flow cytometric analysis of propidium iodide (PI) staining showing cell death after OGD/R. (D, E) Western blot analysis of apoptosis‐related and phosphorylated proteins in HT22 cells following OGD/R. Data are presented as mean ± SEM (*n*  = 3 per group). One‐way ANOVA with Tukey's multiple comparisons test; p‐RBM38, *p =* 0.0256; p‐HIF1α, *p =* 0.0435; p‐p53, *p =* 0.0315; Bcl2/Bax, *p =* 0.0154; Cleaved Caspase‐3, *p =* 0.0247 versus OGD/R‐Vector group. (F) Representative immunofluorescence images of ROS (green) in HT22 cells (scale bar = 30 µm). (G) Quantification of ROS intensity in HT22 cells. Data are mean ± SEM (*n*  = 6 per group). *p* < 0.001 versus OGD/R‐Vector group; one‐way ANOVA with Tukey's multiple comparisons test. (H) Representative immunofluorescence images of ROS (green) in BV2 cells (scale bar = 30 µm). (I) Quantification of ROS intensity in BV2 cells. Data are mean ± SEM (*n*  = 6 per group). *p* < 0.001 versus OGD/R‐Vector group; one‐way ANOVA with Tukey's multiple comparisons test. (J) Representative immunofluorescence images of CD16/32 (pro‐inflammatory marker, red) in BV2 cells (scale bar = 30 µm). (K) Quantification of CD16/32 intensity in BV2 cells. Data are mean ± SEM (*n*  = 6 per group). *p* < 0.001 versus OGD/R‐Vector group; one‐way ANOVA with Tukey's multiple comparisons test. (L) Representative immunofluorescence images of CD206 (anti‐inflammatory marker, green) in BV2 cells (scale bar = 30 µm). (M) Quantification of CD206 intensity in BV2 cells. Data are mean ± SEM (*n*  = 6 per group). *p* < 0.001 versus OGD/R‐Vector group; one‐way ANOVA with Tukey's multiple comparisons test. (N, O) qPCR analysis of pro‐ and anti‐inflammatory cytokine expression (*n* = 3). *p* < 0.001 versus OGD/R‐Vector group; one‐way ANOVA followed by Tukey's multiple comparisons test. ns = not significant, ^*^ *p* < 0.05, ^**^ *p* < 0.01, ^***^ *p* < 0.001. See also Figure S2 and Table S1.

S/T‐P phosphorylation represents a key regulatory mechanism in neuronal signal transduction, yet its pathological role in ischemic brain injury remains undefined [2, 39]. MCAO/R injury triggered significant phosphorylation increases at known GSK3β target sites: RBM38‐S195, HIF1α‐S589, and p53‐S315 in ipsilateral cortex (Figure 2A). Consistently, OGD/R induced a time‐related increase in the phosphorylation of these substrates in both HT22 neurons and BV2 microglia (Figure S2A–D). These findings strongly implicate GSK3β’s proline‐directed kinase activity in hypoxia/reperfusion injury.

OGD/R injury exacerbates cell death, as evidenced by reduced viability (Figure 2B) and increased PI uptake (Figure 2C). Nb.29E9 overexpression significantly attenuated OGD/R‐induced cytotoxicity (Figure 2B,C). Mechanistic analysis revealed that Nb.29E9 restored the Bcl2/Bax ratio and suppressed Cleaved Caspase‐3 levels in both HT22 and BV2 cells (Figure 2D,E and Figure S2E). Furthermore, quantification of intracellular reactive oxygen species (ROS) using DCFH‐DA demonstrated that Nb.29E9 effectively reduced ROS accumulation (Figure 2F–I). Given the critical role of microglial polarization in neuroinflammation, we assessed phenotypic shifts and observed that Nb.29E9 significantly reduced the expression of the pro‐inflammatory marker CD16/32 while increasing that of the anti‐inflammatory marker CD206 in BV2 microglia (Figure 2J–M). Quantitative PCR confirmed that Nb.29E9 markedly downregulated pro‐inflammatory cytokine mRNAs (*IL‐1β*, *IL‐6*, *TNF‐α*) while upregulated anti‐inflammatory cytokines (*IL‐4*, *IL‐10*, *TGF‐β*) (Figure 2N,O).

Collectively, these data demonstrate that Nb.29E9‐mediated inhibition of GSK3β S/T‐P kinase activity provides comprehensive neuroprotection through coordinated suppression of apoptosis, mitigation of oxidative damage, and modulation of neuroimmune responses in the OGD/R model.

### 2.3 A Targeted Fusion Protein, TP‐Nb.29E9, Remodels the Proline‐Directed Phosphoproteome to Promote Neuronal Survival Under Ischemic Stress

Effective neurotherapeutics require dual capabilities: efficient BBB penetration and cell‐type specificity [40]. To achieve this, we engineered a modular fusion protein, TP‐Nb.29E9, by fusing the BBB‐translocating peptide TGN, a cell‐targeting peptide (Tet1 for neurons and MG1 for microglia), and the therapeutic nanobody Nb.29E9  via flexible linkers (Figure S3A) [31, 41]. Purified recombinant TP‐Nb.29E9 exhibited favorable biocompatibility, with cell viability exceeding 91% in both HT22 neurons and BV2 microglia across the tested concentration range (1–10 µm) (Figure S3B). Concentration‐response fitting revealed EC_50_ values of 1.53 µm in HT22 cells and 2.19 µm in BV2 cells (Figure S3C). Accordingly, 5 µm was established as the standardized working concentration for subsequent mechanistic experiments. This dosage ensures near‐maximal pharmacological efficacy while remaining well below the threshold of detectable cytotoxicity, maintaining an optimal safety margin.

**FIGURE 3:**
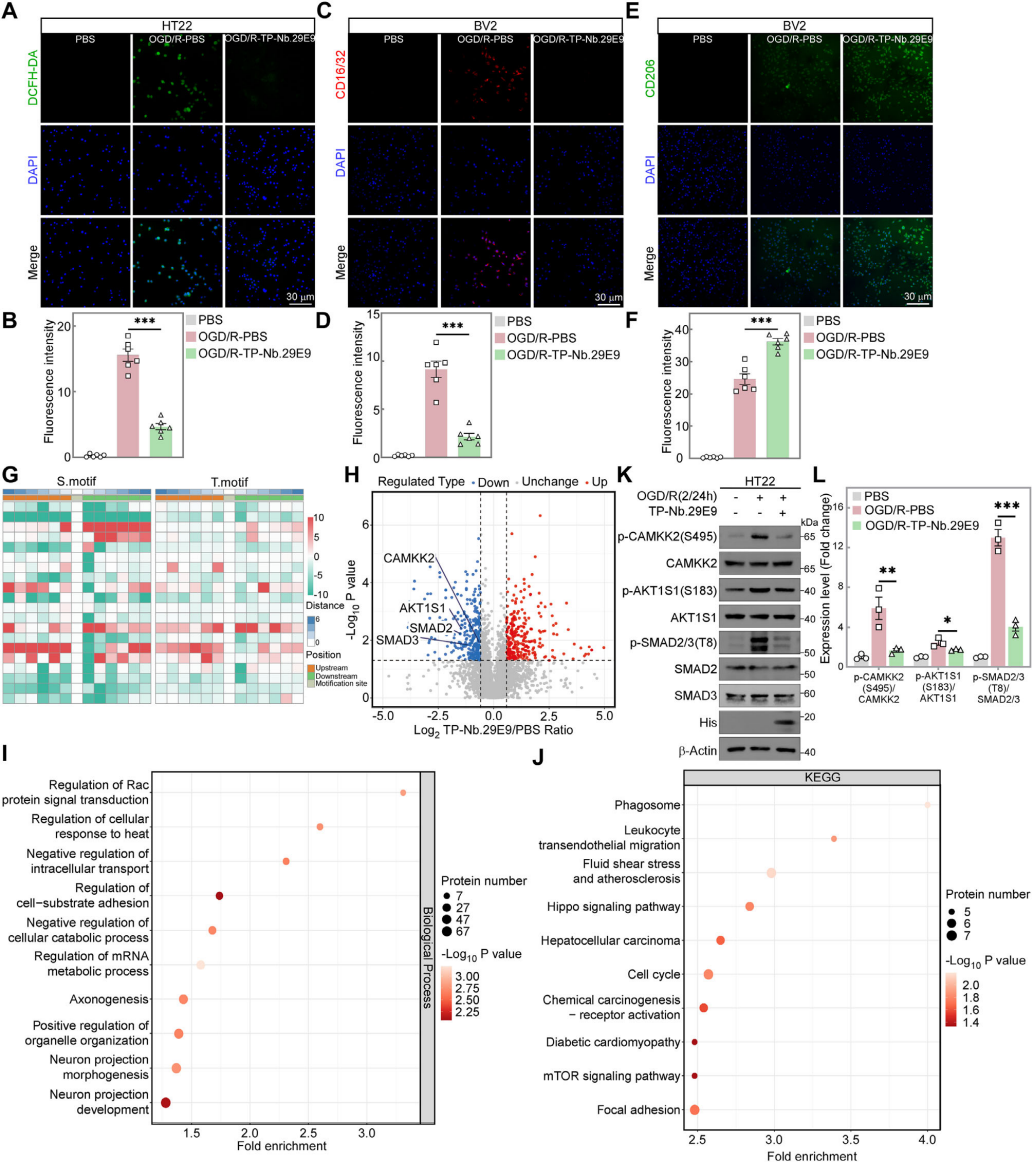
TP‐Nb.29E9 suppresses GSK3β signaling and remodels the phosphoproteome under ischemic stress. (A) Representative immunofluorescence images of ROS (green) in HT22 cells (scale bar = 30 µm). (B) Quantification of ROS intensity in HT22 cells. Data are mean ± SEM (*n*  = 6 per group). *p* < 0.001 vs. OGD/R‐PBS group; one‐way ANOVA with Tukey's multiple comparisons test. (C) Representative immunofluorescence images of CD16/32 (red, pro‐inflammatory marker) in BV2 cells (scale bar = 30 µm). (D) Quantification of CD16/32 intensity in BV2 cells. Data are mean ± SEM (*n*  = 6 per group). *p* < 0.001 versus OGD/R‐PBS group; one‐way ANOVA with Tukey's multiple comparisons test. (E) Representative immunofluorescence images of CD206 (green, anti‐inflammatory) in BV2 cells (scale bar = 30 µm). (F) Quantification of CD206 (green, anti‐inflammatory marker) in BV2 cells. Data are mean ± SEM (*n*  = 6 per group). *p* < 0.001 versus OGD/R‐PBS group; one‐way ANOVA with Tukey's multiple comparisons test. (G) Motif analysis of 4993 quantified phosphosites highlighting enrichment of S/T‐P motifs. (H) Volcano plot of 577 significantly regulated phosphosites in HT22 cells upon TP‐Nb.29E9 treatment. (I, J) GO (I) and KEGG (J) enrichment analysis of pathways related to neuronal survival and synaptic function. (K) Western blot analysis of SMAD2/3 (Thr8), CAMKK2 (Ser495), and AKT1S1 (Ser183) phosphorylation. (L) *p* values versus OGD/R‐PBS group: p‐CAMKK2, *p =* 0.0073; p‐AKT1S1, *p =* 0.0288; p‐SMAD2/3, *p* < 0.001; one‐way ANOVA with Tukey's multiple comparisons test. ns = not significant, ^*^ *p* < 0.05, ^**^ *p* < 0.01, ^***^ *p* < 0.001. See also Figure S3 and Figure S4.

Live‐cell imaging revealed target‐specific accumulation of TP‐Nb.29E9 in HT22 neurons and BV2 microglia (Figure S3D,E), and western blot confirmed fusion protein expression and stability (Figure S3F). TP‐Nb.29E9 effectively inhibited GSK3β S/T‐P kinase activity, as shown by reduced phosphorylation of p‐RBM38 (S195) in both cell types (Figure S3G). Under OGD/R conditions, TP‐Nb.29E9 conferred comprehensive neuroprotection, enhancing cell viability (Figure S3H,I), suppressing Cleaved Caspase‐3 (Figure S3G), and reducing ROS accumulation (Figure 3A,B and Figure S3J,K). Concurrently, it reversed pro‐inflammatory microglial polarization in BV2 cells, decreasing CD16/32 expression and increasing CD206 expression (Figure 3C–F). qPCR analysis confirmed that TP‐Nb.29E9 significantly downregulated pro‐inflammatory cytokine mRNAs (*IL‐1β*, *IL‐6*, *TNF‐α*) while upregulating anti‐inflammatory cytokines (*TGF‐β, IL‐10, IL‐4*) (Figure S3L,M).

To systematically characterize the mechanisms of Nb.29E9, we performed quantitative phosphoproteomics in OGD/R‐treated HT22 cells. Among 4,993 identified phosphosites, Motif‐X analysis revealed significant enrichment of S/T‐P phosphorylation motifs (Figure 3G and Figure S4A), indicating a distinct eIF4E2‐GSK3β pathway signature divergent from canonical primed‐substrate regulation. Analysis of 577 significantly regulated phosphosites (247 downregulated; Figure 3H) showed GO enrichment in neuronal survival, synaptic regulation, and stress response (Figure 3I and Figure S4B,C), while KEGG mapping identified key pathways controlling apoptosis, synaptic homeostasis, and metabolic adaptation (Figure 3J).

Validation studies demonstrated that TP‐Nb.29E9 significantly suppressed S/T‐P phosphorylation at several critical signaling nodes, including the TGFβ mediators SMAD2/3 (Thr8), AMPK kinase CAMKK2 (Ser495), and mTORC1 regulator PRAS40/AKT1S1 (Ser183) (Figure 3H–L) [42, 43, 44]. Collectively, these findings establish that TP‐Nb.29E9 mediates cell‐specific neuroprotection and anti‐inflammatory microglial reprogramming through selective GSK3β inhibition, remodeling phosphosignaling networks to restore neuronal homeostasis under ischemic stress.

### 2.4 An MMP‐9‐Responsive Ferritin Nanoparticle Enables Efficient Brain‐Targeted Delivery of TP‐Nb.29E9

**FIGURE 4:**
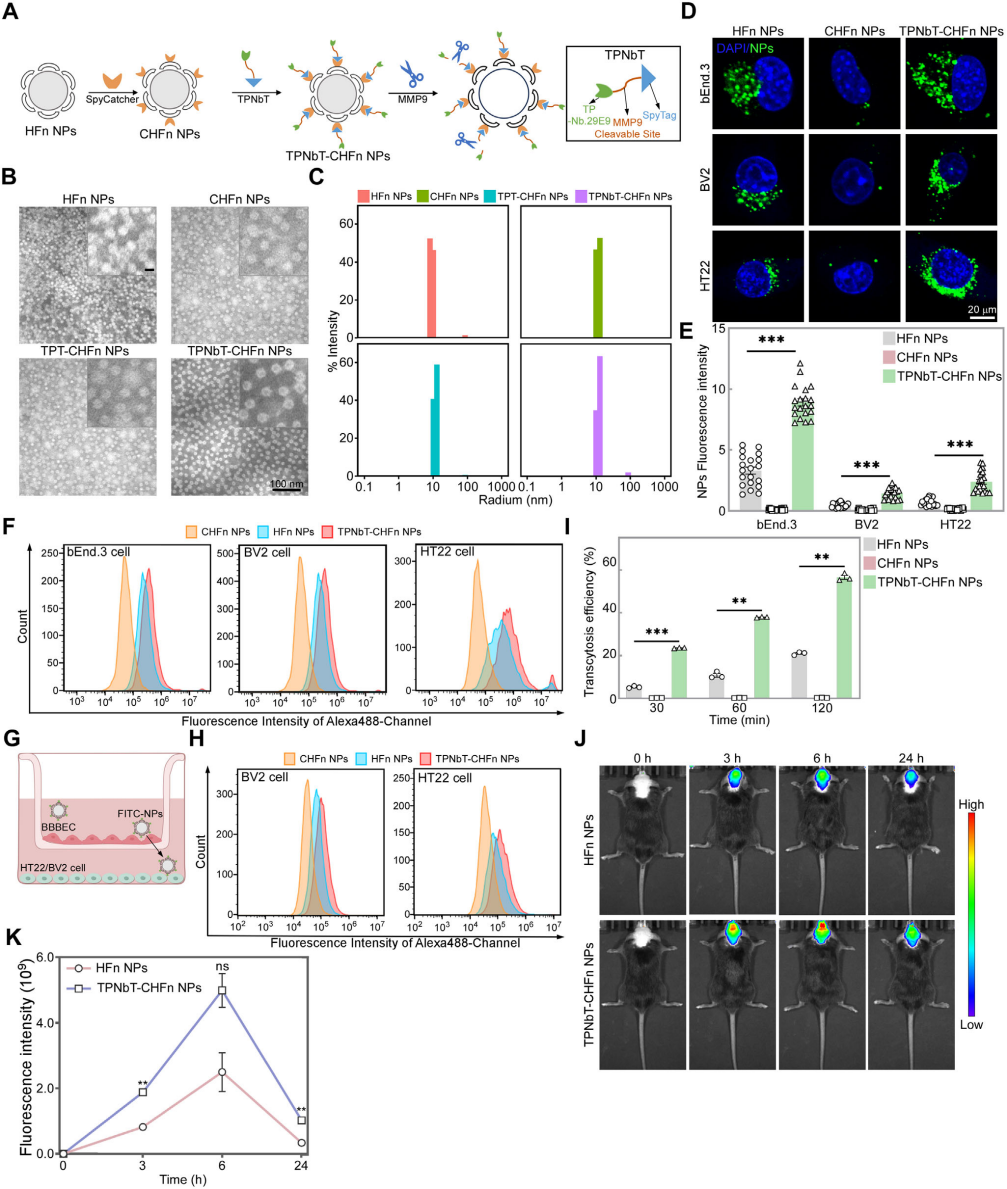
Engineering a stroke‐responsive, BBB‐penetrant nanocarrier for brain delivery of Nb.29E9. (A) Schematic of the pre‐assembled CHFn nanoparticle conjugated to an MMP‐9‐cleavable TPNbT module via SpyTag/SpyCatcher chemistry. (B, C) TEM (B) and DLS (C) analysis confirm uniform morphology and successful conjugation, as evidenced by increased hydrodynamic diameter (scale bar = 100 nm). (D, E) Representative immunofluorescence images (D) and quantification (E) of TPNbT‐CHFn NP uptake in bEnd.3, HT22, and BV2 cells. For quantification (E), mean fluorescence intensity was determined from approximately 20 cells per biological replicate; data are mean ± SEM (*n* = 3 biological replicates). *p* < 0.001 versus HFn NPs group; one‐way ANOVA with Tukey's multiple comparisons test. Scale bar = 20 µm. (F) Flow cytometric analysis of cellular uptake in the three cell lines. (G–I) In vitro BBB model demonstrates higher transcytosis efficiency and subsequent uptake in neurons and microglia (*n* = 3 per group). Two‐way repeated measures ANOVA: Group × Time interaction, *p* < 0.001 versus HFn NPs group. *p* < 0.001 at 30 min, *p =* 0.0072 at 60 min, *p =* 0.0012 at 120 min, TPNbT‐CHFn NPs versus HFn NPs, individual time‐point comparisons. (J) Representative serial in vivo fluorescence images of a mouse. (K) Quantification of in vivo fluorescence intensity (*n =* 3 mice per group). Two‐way repeated measures ANOVA: Group × Time interaction, *p =* 0.0022 versus HFn NPs group. *p =* 0.0092 at 3 h, *p =* 0.0995 at 6 h, *p =* 0.0027 at 24 h, TPNbT‐CHFn NPs vs. HFn NPs, individual time‐point comparisons. ns = not significant, ^*^ *p* < 0.05, ^**^ *p* < 0.01, ^***^ *p* < 0.001. See also Figure S5.

Building on the therapeutic promise of TP‐Nb.29E9 while addressing its stroke delivery limitations, we engineered a stimuli‐responsive ferritin nanoparticle (NP) system using SpyTag/SpyCatcher bioorthogonal conjugation [45]. The CHFn nanoparticle was generated by fusing SpyCatcher to the N‐terminus of human heavy‐chain ferritin (HFn). TP‐Nb.29E9 was modified with a C‐terminal SpyTag via an MMP‐9‐cleavable linker (GGGSGLGRMGLPGK), creating TPNbT (Figure S5A). Functional nanoparticles assembled through covalent SpyTag‐SpyCatcher conjugation between TPNbT and CHFn (Figure S5A). Control TPT‐CHFn NPs lacking the therapeutic nanobody domain were prepared using SpyTag‐tagged targeting peptide (TPT) (Figure S5A). To prevent spatial interference of direct nanobody fusion with HFn's structural assembly, we implemented a “pre‐assembly and post‐modification” strategy: complete CHFn NPs formed prior to surface functionalization (Figure 4A). All recombinant proteins expressed efficiently in soluble form (TPNbT: 23.7 kDa; CHFn: 38.9 kDa; Figure S5B) with high conjugation efficiency (TPNbT‐CHFn: 62.6 kDa; Figure S5B). Western blot confirmed MMP‐9 specifically cleaved TPNbT‐CHFn NPs to release 20–25 kDa active TP‐Nb.29E9 (Figure S5C). Transmission electron microscopy (TEM) revealed all NPs maintained uniform, hollow spherical morphology (Figure 4B). Dynamic light scattering (DLS) demonstrated narrow size distributions and monodispersity, with hydrodynamic diameters progressively increasing from 10.17 nm (HFn NPs) to 13.37 nm (TPNbT‐CHFn NPs), consistent with successful surface modifications (Figure 4C).

Cellular targeting performance was evaluated by binding/uptake analysis. Fluorescence microscopy and flow cytometry revealed TPNbT‐CHFn NP uptake reached 45.9% in brain microvascular endothelial cells (bEnd.3), 39.4% in microglia (BV2), and 65.0% in neurons (HT22) (Figure 4D–F), representing 1.5‐ to 3.2‐fold enhancements over HFn NPs. In an in vitro BBB model, TPNbT‐CHFn NPs exhibited 1.9‐fold higher transcytosis efficiency than HFn NPs (Figure 4G,H), with subsequent basolateral uptake rates of 38.2% by HT22 neurons and 30.4% by BV2 microglia (Figure 4I). Notably, CHFn lost native HFn targeting capacity due to SpyCatcher surface modification (Figure 4G–I).

In vivo fluorescence imaging revealed markedly enhanced brain accumulation of TPNbT‑CHFn NPs compared to HFn NPs, with an approximately 2‑fold higher signal at 3 h post‑injection and peak accumulation at 6 h (Figure 4J,K). Ex vivo imaging of isolated brains further corroborated the superior brain enrichment of TPNbT‑CHFn NPs relative to HFn NPs (Figure S5D,E). Moreover, co‐staining of brain sections for the His‐tagged nanobody and cell‐type markers revealed that TPNbT‐CHFn NPs accumulated in significantly more NeuN^+^ neurons and Iba1^+^ microglia than HFn NPs (Figure S5F–I). Hematoxylin and eosin (H&E) staining of major organs (heart, liver, spleen, lungs, kidneys, brain) following intravenous administration (50 mg/kg) revealed no pathological alterations (Figure S5J), confirming excellent in vivo biocompatibility.

Collectively, these data demonstrate the engineered TPNbT‐CHFn system effectively accomplishes targeted, blood‐brain barrier‐penetrant nanobody delivery with cell‐type specificity.

### 2.5 TPNbT‐CHFn Nanoparticles Alleviate Subacute Neuronal Injury and Neuroinflammation After Ischemic Stroke

**FIGURE 5:**
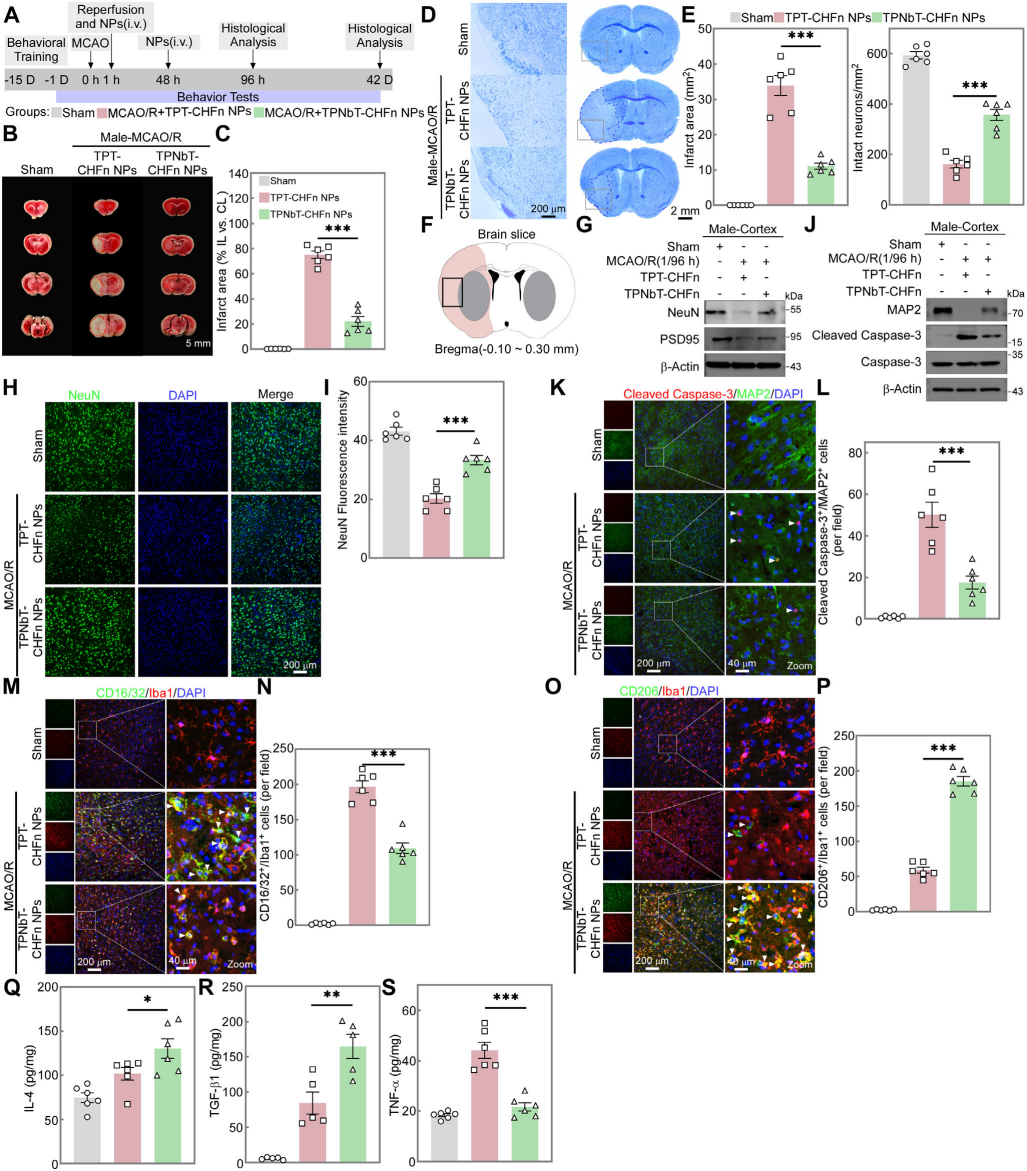
TPNbT‐CHFn NPs protect neurons and mitigate neuroinflammation in subacute MCAO/R. (A) Schematic illustration of the treatment regimen. MCAO/R mice were injected intravenously with TPT‐CHFn NPs or TPNbT‐CHFn NPs immediately upon reperfusion (0 h) and again at 48 h post‐reperfusion. (B) Representative TTC‐stained coronal brain sections at 96 h post‐reperfusion from Sham, MCAO/R + TPT‐CHFn NPs, and MCAO/R + TPNbT‐CHFn NPs groups. (C) Quantification of cerebral infarct volume. Data are mean ± SEM (*n* = 6 mice per group). *p* < 0.001 versus TPT‐CHFn NPs group; one‐way ANOVA followed by Tukey's multiple‐comparisons test. (D) Representative Nissl staining of the infarct cortical (scale bar = 200 µm). (E) Quantification of intact neurons and lesion‐associated tissue damage based on Nissl staining. Data are mean ± SEM (*n* = 6 mice per group). *p* < 0.001 vs. TPT‐CHFn NPs group; one‐way ANOVA followed by Tukey's multiple‐comparisons test. (F) Schematic coronal section (bregma ‐0.10 to +0.30 mm). Pink regions indicate ischemic injury; boxed areas denote regions of interest used for histological analyses. (G) Western blot analysis of NeuN and PSD95 protein levels in ischemic cortical tissue. (H) Representative immunofluorescence staining for NeuN (green) in the infarct regions (scale bar = 200 µm). (I) Quantification of NeuN fluorescence intensity. Data are mean ± SEM (*n* = 6 mice per group). *p* < 0.001 versus MCAO/R+ TPT‐CHFn NPs group; one‐way ANOVA followed by Tukey's multiple‐comparisons test. (J) Western blot analysis of MAP2, Cleaved Caspase‐3 and total Caspase‐3 in ischemic cortical tissue. (K) Representative immunofluorescence staining for MAP2 (green) and Cleaved Caspase‐3 (red). Arrows indicate MAP2^+^/Cleaved Caspase‐3^+^ colocalized cells (scale bar = 40 µm). (L) Quantification of MAP2^+^/Cleaved Caspase‐3^+^ cells per field. Data are mean ± SEM (*n* = 6 mice per group). *p* < 0.001 versus MCAO/R + TPT‐CHFn NPs group; one‐way ANOVA followed by Tukey's multiple‐comparisons test. (M) Representative immunofluorescence staining for CD16/32 (green) and Iba1 (red). Arrows indicate CD16/32^+^/Iba1^+^ pro‐inflammatory microglia (scale bar = 40 µm). (N) Quantification of CD16/32^+^/Iba1^+^ cells per field. Data are mean ± SEM (*n* = 6 mice per group). *p* < 0.001 vs. MCAO/R + TPT‐CHFn NPs group; one‐way ANOVA followed by Tukey's multiple‐comparisons test. (O) Representative immunofluorescence staining for CD206 (green) and Iba1 (red). Arrows indicate CD206^+^/Iba1^+^ anti‐inflammatory microglia (scale bar = 40 µm). (P) Quantification of CD206^+^/Iba1^+^ cells per field. Data are mean ± SEM (*n* = 6 mice per group). *p* < 0.001 versus MCAO/R+ TPT‐CHFn NPs group; one‐way ANOVA followed by Tukey's multiple‐comparisons test. (Q–S) ELISA analysis of IL‐4 (Q), TGF‐β1 (R) and TNF‐α (S) levels in ischemic cortical tissue. Data are mean ± SEM (*n* = 5–6 mice per group). IL‐4: *p =* 0.0491, TGF‐β1: *p =* 0.0022, TNF‐α: *p* < 0.001, vs. MCAO/R + TPT‐CHFn NPs group; one‐way ANOVA followed by Tukey's multiple comparisons test. ns = not significant, ^*^ *p* < 0.05, ^**^ *p* < 0.01, ^***^ *p* < 0.001. See also Figure S6 and Table S1.

To evaluate the therapeutic efficacy of TPNbT‐CHFn NPs in ischemia–reperfusion injury, a transient MCAO/R model was established and a multiple‐dose regimen was applied. TPNbT‐CHFn NPs (10 mg/kg, 200 µL) were intravenously administered at the onset of reperfusion (0 h) and again at 48 h, while control mice received an equivalent dose of TPT‐CHFn NPs (Figure 5A). Short‐term therapeutic effects were assessed in both male and female mice.

TTC staining demonstrated that TPNbT‐CHFn NPs markedly reduced cerebral infarct volume in both sexes compared with the control group (male: from 66.67% to 23.14%; female: from 56.84% to 15.55%), indicating comparable acute neuroprotection across sexes (Figure 5B,C and Figure S6A,B). Consistent with this structural protection, TPNbT‐CHFn NPs significantly improved neurological performance over 4 days following reperfusion in both male and female mice, as assessed by the modified neurological severity score (mNSS), a composite evaluation of motor, sensory, reflex, and balance functions (Figure S6C,D).

Histological analyses further corroborated these protective effects. Nissl staining revealed preserved neuronal density and structural integrity in the ischemic penumbra (Figure 5D,E). Neuronal survival was additionally confirmed by increased NeuN (RBFOX3) expression, as shown by Western blotting, immunostaining, and qPCR analyses (Figure 5G–I and Figure S6E,F). Moreover, reduced Cleaved Caspase‐3 levels together with restored MAP2 expression indicated effective suppression of neuronal apoptosis (Figure 5J–L and Figure S6G). Synaptic integrity was also preserved, as evidenced by upregulated PSD95 at both the mRNA and protein levels at 96 h post‐treatment (Figure 5G and Figure S6E,F).

We next examined whether these neuroprotective effects were accompanied by alleviation of oxidative and endoplasmic reticulum (ER) stress. ROS staining showed a marked reduction in reactive oxygen species levels following TPNbT‐CHFn NP treatment (Figure S6H,I). In parallel, the nanoparticles upregulated antioxidant genes (*SOD2* and *NQO1*), downregulated the ER stress marker *CHOP* (Figure S6J), decreased malondialdehyde (MDA) content, and increased superoxide dismutase (SOD) activity (Figure S6K,L), collectively indicating improved redox homeostasis.

Given the critical contribution of post‐ischemic neuroinflammation to secondary brain injury, microglial polarization was subsequently assessed. Immunofluorescence staining showed that TPNbT‐CHFn NPs decreased the proportion of CD16/32^+^ pro‐inflammatory microglia and increased CD206^+^ anti‐inflammatory microglia in the ipsilateral cortex (Figure 5M–P). These findings were supported by ELISA and qPCR analyses, which demonstrated reduced expression of M1‐associated markers (CD32 and CD86) and TNF‐α, alongside increased levels of anti‐inflammatory mediators (IL‐4 and TGF‐β) at both the protein and transcript levels (Figure 5Q–S and Figure S6M,N).

At the signaling level, TPNbT‐CHFn NPs suppressed MCAO/R‐induced S/T‐P phosphorylation of multiple putative GSK3β downstream targets, including CAMKK2 (S495), AKT1S1 (S183), SMAD2/3 (T8), and RBM38 (S195), in both male and female mice. These findings are consistent with the phosphoproteomic profiling results (Figure S6O,P).

Collectively, these results demonstrate that TPNbT‐CHFn NPs provide robust acute neuroprotection after ischemia‐reperfusion injury by reducing infarct burden, improving neurological outcomes, preserving neuronal and synaptic integrity, and attenuating oxidative stress and neuroinflammation.

### 2.6 TPNbT‐CHFn Nanoparticles Promote Long‐Term Functional Recovery and Neurovascular Repair

To investigate the long‐term therapeutic effects of TPNbT‐CHFn NPs following ischemia‐reperfusion injury, neurological function was assessed using a battery of behavioral tests, including the beam balance test, horizontal ladder rung walking test, and rotarod test. These long‐term evaluations were performed in male mice.

**FIGURE 6:**
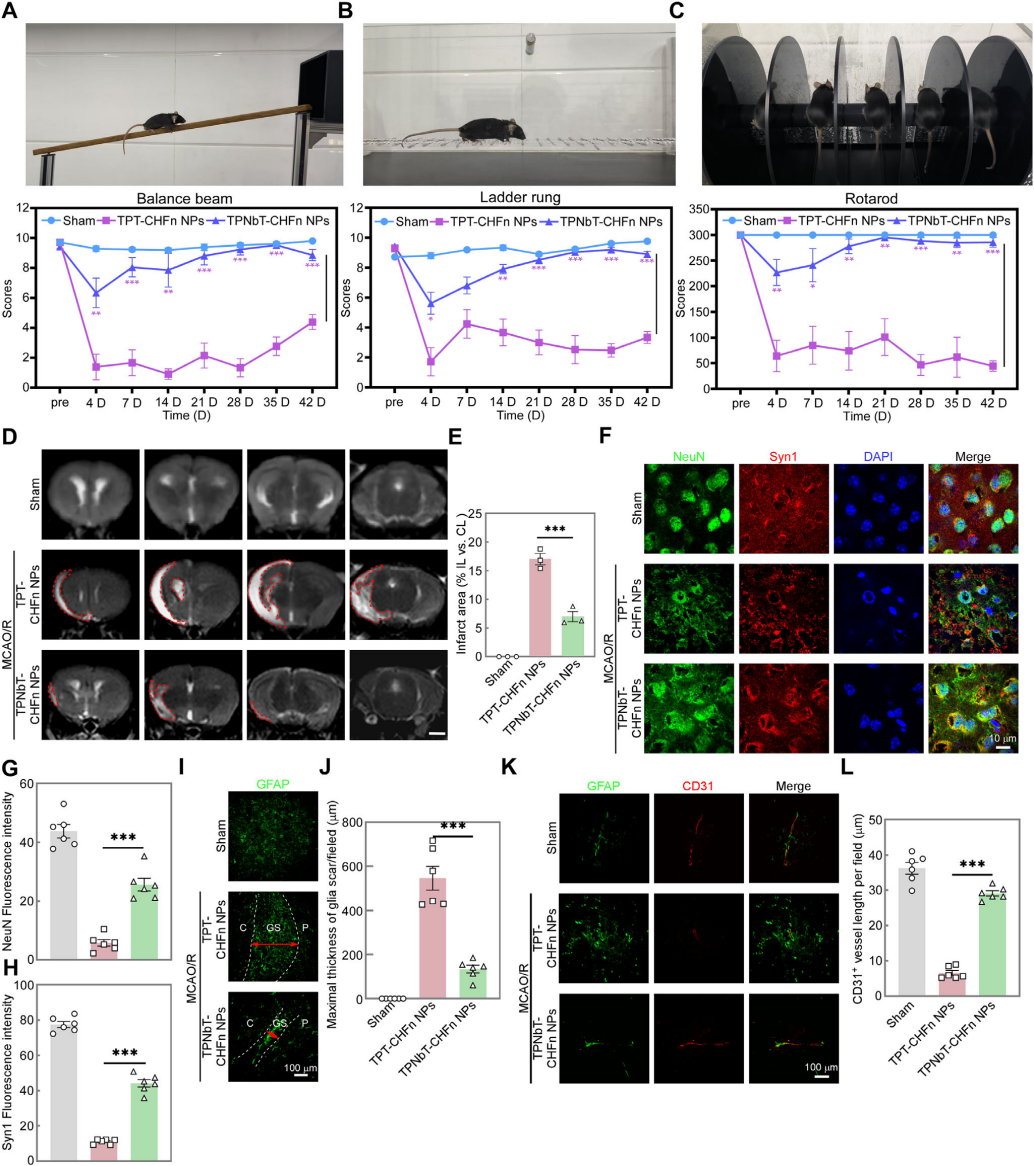
TPNbT‐CHFn NPs promote long‐term neurovascular repair and functional recovery after MCAO/R. (A–C) Longitudinal behavioral assessments using the balance beam test (A), horizontal ladder rung walking test (B), and rotarod test (C) performed at baseline (pre) and over 42 days post‐MCAO/R. TPNbT‐CHFn NP‐treated mice exhibited significantly better functional outcomes compared with the MCAO/R + TPT‐CHFn NPs group. Data are mean ± SEM (*n* = 7 mice per group). Balance beam: *p* < 0.001; Ladder rung: *p* < 0.001; Rotarod: *p* < 0.001, vs. the MCAO/R+ TPT‐CHFn NPs group; two‐way repeated‐measures ANOVA followed by post hoc multiple‐comparisons tests. (D) Representative MRI images of Sham, MCAO/R + TPT‐CHFn NPs, and MCAO/R+ TPNbT‐CHFn NPs groups at 42 days post‐reperfusion. Four consecutive coronal sections are shown for each group (scale bar = 2 mm). (E) Quantification of total infarct volume based on MRI analysis. Data are mean ± SEM (*n =* 3 mice per group). *p* < 0.001 vs. MCAO/R + TPT‐CHFn NPs group; one‐way ANOVA followed by Tukey's multiple comparisons test. (F) Representative immunofluorescence staining for NeuN (green) and Syn1 (red) in the infarct regions (scale bar = 10 µm). (G, H) Quantification of NeuN (G) and Syn1 (H) signal intensity. Data are mean ± SEM (*n* = 6 mice per group). *p* < 0.001 versus MCAO/R + TPT‐CHFn NPs group; one‐way ANOVA followed by Tukey's multiple comparisons test. (I) Representative immunofluorescence staining for GFAP (green) showing reactive astrogliosis in the lesional tissue (scale bar = 100 µm). (J) Quantification of GFAP‐positive area in the infarct region. Data are mean ± SEM (*n* = 6 mice per group). *p* < 0.001 compared with the MCAO/R + TPT‐CHFn NPs group; one‐way ANOVA followed by Tukey's multiple comparisons test. (K) Representative immunofluorescence staining for GFAP (green) and CD31 (red) showing neurovascular architecture in the infarct regions (scale bar = 100 µm). (L) Quantification of CD31‐positive vessel length per field. Data are mean ± SEM (*n* = 6 mice per group). *p* < 0.001 compared with the MCAO/R + TPT‐CHFn NPs group; one‐way ANOVA followed by Tukey's multiple‐comparisons test. ns = not significant, ^*^ *p* < 0.05, ^**^ *p* < 0.01, ^***^ *p* < 0.001. See also Figure S7.

Compared to the sham group, MCAO/R mice treated with TPT‐CHFn NPs exhibited pronounced neurological deficits across all tests (Figure 6A–C). In contrast, TPNbT‐CHFn NPs treatment significantly improved functional performance over time (Figure 6A–C). At 6 weeks post‐reperfusion, magnetic resonance imaging (MRI) revealed a marked reduction in infarct volume in TPNbT‐CHFn NPs‐treated mice compared with controls (Figure 6D,E), which was further supported by morphological and gravimetric analyses (Figure S7A–C).

Histological and immunofluorescence analyses demonstrated improved neuronal status following treatment. Nissl staining, together with NeuN, Synapsin‐1 (Syn1), and PSD95 immunostaining, indicated enhanced neuronal survival and synaptic integrity in the TPNbT‐CHFn NPs group (Figure 6F–H and Figure S7D–G). Glial scar formation was next evaluated in the chronic phase. TPNbT‐CHFn NPs treatment significantly reduced GFAP‐positive reactive astrogliosis compared with controls (Figure 6I,J). We further examined the neurovascular unit (NVU) at 6 weeks post‐injury. Immunofluorescence analysis showed that CD31‐labeled vascular structures were more continuous and less fragmented, and GFAP‐positive astrocytic processes exhibited improved association with the vascular wall in TPNbT‐CHFn NPs‐treated mice, indicating restoration of neurovascular architecture (Figure 6K,L).

Collectively, these findings demonstrate that TPNbT‐CHFn NPs promote sustained functional recovery and structural remodeling of the neurovascular unit following ischemic injury.

## 3 Discussion

Ischemic stroke remains a major therapeutic challenge, with GSK3β emerging as a pivotal yet complex target due to its dual kinase activities and central role in neuronal survival and death pathways [9]. Although broadly inhibiting GSK3β can confer neuroprotection, conventional ATP‐competitive inhibitors cannot distinguish between its primed‐substrate and proline‐directed activities, thereby limiting therapeutic precision and clinical translation [2, 46, 47]. In this study, we address this limitation by developing an activity‐specific nanobody, Nb.29E9, which selectively targets the S/T‐P kinase module of GSK3β. By inhibiting this pathogenic activity while preserving physiological primed‐substrate phosphorylation, our findings identify proline‐directed phosphorylation as a critical driver of ischemic neurodegeneration and establish a mechanism‐guided therapeutic strategy.

Nanobody‐based strategies for kinase modulation have attracted increasing attention; however, most reported approaches primarily rely on direct inhibition of kinase catalytic activity or interference with protein stability, which often lack substrate selectivity [48, 49]. In contrast, Nb.29E9 selectively disrupts the regulatory interaction between GSK3β and eIF4E2, thereby targeting a context‐dependent phosphorylation program without competing for the ATP‐binding pocket. This interface‐directed mode of action enables activity‐specific modulation and may reduce off‐target effects associated with global kinase inhibition [37, 38]. Given that many kinases depend on scaffold or adaptor proteins to define substrate specificity, this strategy may provide a broadly applicable framework for achieving selective regulation of proline‐directed kinases such as CDKs, MAPKs, and JNKs [50, 51, 52].

Consistent with this mechanism, our phosphoproteomic analyses indicate that Nb.29E9 induces coordinated remodeling of proline‐directed phosphorylation networks. Key signaling nodes, including SMAD2/3, CAMKK2, and AKT1S1, were selectively modulated, suggesting that GSK3β S/T‐P activity functions as a central integrator of multiple pathological processes in ischemic injury [43, 44, 53]. Through simultaneous regulation of pathways associated with apoptosis, oxidative stress, and neuroinflammation, selective inhibition of this activity confers a multi‐faceted neuroprotective effect. While these findings provide a systems‐level perspective, further studies will be required to delineate the direct substrates and causal relationships underlying these effects.

From a translational perspective, the clinical application of nanobody‐based therapeutics in CNS disorders is largely constrained by delivery barriers, particularly BBB penetration [54]. To address this challenge, we developed a ferritin‐based nanoparticle platform that facilitates efficient transport of Nb.29E9 into the brain and enables its release within the ischemic microenvironment [31, 45, 55]. This delivery strategy enhances local bioavailability and target engagement, providing a practical solution for translating mechanism‐specific biologics into the central nervous system. Notably, the therapeutic efficacy observed in the acute phase was consistent between male and female mice, supporting the robustness of this approach across sexes.

Despite these promising findings, several limitations should be acknowledged. The MCAO/R model, while widely used, does not fully recapitulate the clinical heterogeneity of human stroke, particularly in the presence of comorbidities such as hypertension or diabetes [56, 57]. In addition, although Nb.29E9 exhibits high specificity toward the eIF4E2‐regulated S/T‐P activity of GSK3β, its broader impact on systemic signaling networks remains to be further evaluated. Finally, optimization of dosing regimens, pharmacokinetics, and long‐term safety will be essential for future clinical translation [58].

## 4 Conclusion

This study identifies GSK3β‐driven proline‐directed phosphorylation as a critical driver of ischemic neurodegeneration and demonstrates that activity‐selective modulation of this axis is both feasible and therapeutically effective. To achieve this, we developed Nb.29E9, an interface‐directed nanobody that selectively suppresses pathological S/T‐P phosphorylation while preserving physiological signaling, and delivered it across the blood‐brain barrier using an ischemia‐responsive ferritin nanoplatform. Systemic administration of this platform reduced ischemic injury and improved long‐term neurological recovery. These findings not only highlight the distinct dual kinase activities of GSK3β but also establish regulatory‐interface targeting as an effective conceptual framework for precision modulation of disease‐associated phosphorylation programs in neurological disorders.

## 5 Methods

The phospho‐specific antibodies p‐RBM38 was generated by immunizing rabbits with the synthetic phospho‐peptide span serine195 [187YDQYPYAAS(p)PAT198] [14]. The RBM38 antibodies were generated by immunizing rabbits with the peptide [195SPATAASFVGYS206] [14]. The phospho‐specific p‐HIF1α(S589) antibodies were generated by immunizing rabbits with the synthetic phospho‐peptide span serine 589 [584ESSSAS(p)PES592] [14]. Table 1.

**TABLE 1:** Key resources table.

Reagent or resource	Source	Identifier
Antibodies		
Caspase‐3	Proteintech	Cat#66470‐2‐Ig
Cleaved Caspase‐3	BD biosciences	Cat#559565
CD16/32	BD biosciences	Cat#553142
CD206	Cell Signaling Technology	Cat#24595
FLAG‐Tag	Sigma–Aldrich	Cat#F4799
GST‐Tag	ABclonal	Cat#AE001
His‐Tag	ABclonal	Cat#AE003
HRP Goat Anti‐Rabbit IgG	ABclonal	Cat#AS014
HRP Goat Anti‐Mouse IgG	ABclonal	Cat#AS003
GSK3β	Santa Cruz Biotechnology	Cat#sc‐81462
HIF1α	Abcam	Cat#ab308433
p53	Abcam	Cat#ab26
p‐p53 (S315)	Cell Signaling Technology	Cat#2528
Bcl2	Abcam	Cat#ab32124
Bax	Abcam	Cat#ab32503
β‐Actin	Abclonal	Cat# AC004
GFAP	Cell Signaling Technology	Cat#3670S
CD31	Abcam	Cat#ab24590
Syn1	Proteintech	Cat#20258‐1‐AP
Iba1	Abcam	Cat#Ab283319
MAP2	Proteintech	Cat#67015‐1‐lg
NeuN	Abcam	Cat#ab177487
PSD95	Proteintech	Cat#20665‐1‐AP
SMAD2	Proteintech	Cat#83841‐2‐RR
SMAD3	Proteintech	Cat#66516‐1‐Ig
CAMKK2	Proteintech	Cat#11549‐1‐AP
AKT1S1	Proteintech	Cat#21097‐1‐AP
p‐AKT1S1 (Ser183)	Affinity	Cat#AF2386
p‐CAMKK1/2 (Ser458/Ser495)	Affinity	Cat#AF8136
p‐SMAD2/3 (Thr8)	Yeasen	Cat#31132ES50
Goat anti‐Mouse IgG 568	Invitrogen	Cat#A‐10037
Goat anti‐ Rabbit IgG 568	Invitrogen	Cat#A‐11036
Goat anti‐Mouse IgG 488	Invitrogen	Cat#A‐11029
Goat Anti‐Rabbit IgG H&L (FITC)	Abcam	Cat#ab6717
Goat Anti‐Mouse IgG H&L (Alexa Fluor 647)	Abcam	Cat#ab150115
Chemicals, peptides, and recombinant proteins		
DCFH‐DA	Sigma	Cat#2044‐85‐1
MMP‐9	MCE	Cat#HY‐P73807
APMP	MCE	Cat#HY‐148905
FITC‐NHS	Yeasen	Cat#3326‐32‐7
Cy5.5	MCE	Cat#HY‐D0924
EDC‐HCl	MCE	Cat#HY‐D0178
TTC	Solarbio	Cat#298‐96‐4
OCT compound	SAKURA	N/A
10% formalin	Coolaber	Cat#SL1560
4% paraformaldehyde	Biosharp	Cat#BL539A
Triton X‐100	Biofroxx	Cat#1139 ML100
Nissl Stain Solution	Solarbio	Cat#G1434
RIPA lysis buffer	Biosharp	Cat#BL504A
Isoflurane	RWD	Cat#R510‐22‐10
ECL Enhanced Kit	Abclonal	Cat#RM00021
Critical commercial assays		
ClonExpress II MultiS One‐Step Cloning Kit	Vazyme	Cat#C113‐01
BCA Protein Assay Kit	Bio‐Rad	Cat#5000002
PrimeScript RT reagent kit	Takara	Cat#RR047A
ChamQ Universal SYBR qPCR Master Mix	Vazyme	Cat#Q711‐03
CCK8	Beyotime	Cat#C0038
BeyoFC Propidium Iodide Staining Solution (50X)	Beyotime	Cat#C1734
ElaBoX ^TM^ Mouse TNF‐α ELISA Kit	Solarbio	Cat#SEKM‐0034
ElaBoX ^TM^ Mouse TGF‐β1 ELISA Kit	Solarbio	Cat#SEKM‐0035
ElaBoX ^TM^ Mouse IL‐4 ELISA Kit	Solarbio	Cat#SEKM‐0005
H&E Staining Kit	Solarbio	Cat#G1120
Deposited data		
protein profile data in this manuscript	PRIDE	PRIDE: PXD067942
Experimental models: Cell lines		
Mouse: BV2 cells	Procell Life Science & Technology Co., Ltd	Cat#CL‐0493
Mouse: HT22 cells	Procell Life Science & Technology Co., Ltd	Cat#CL‐0697
Human: HEK‐293T3	ATCC	Cat#CRL‐11268
Human: bEnd.3	Xiangru Wang, Huazhong Agriculture University	Cat# CRL‐2299
Experimental models: Organisms/strains		
Mouse: C57BL/6	Huazhong Agriculture University Laboratory Animal Center	N/A
Oligonucleotides		
See Table S1 for the qRT‐PCR primer list.	This paper	N/A
Software and algorithms		
Prism	GraphPad Software	https://www.graphpad.com/
IMAGE J	National Institutes of Health	https://imagej.nih.gov/ij
LAS X	Leica	https://www.leica‐microsystems.com/products/microscopesoftware/p/leica‐las‐x‐ls/downloads/
HADDOCK 2.4	Bonvinlab	https://rascar.science.uu.nl/haddock2.4/
Living Image 4.3	Perkin Elmer	https://www.perkinelmer.com
Gen5	Biotek	https://www.biotek.com

## 6 Experimental Model and Study Participant Details

### 6.1 Cell Lines

Human embryonic kidney cells (HEK‐293T, CRL‐11268, ATCC), mouse microglial cells (BV2, CL‐0493, Procell), mouse hippocampal neuronal cells (HT22, CL‐0697, Procell), and mouse brain endothelial cells (bEnd.3, CRL‐2299, ATCC, kindly provided by Dr. Xiangru Wang, Huazhong Agricultural University) were cultured in Dulbecco's Modified Eagle Medium (DMEM, C11995500BT, Gibco) supplemented with 10% (v/v) fetal bovine serum (FBS, 16000–044, Gibco) and 1% (v/v) penicillin/streptomycin solution (ST488, Beyotime). All cell lines were maintained at 37°C in a humidified atmosphere containing 5% CO_2_ and were routinely tested to confirm the absence of mycoplasma and bacterial contamination.

### 6.2 Mice

Both male and female C57BL/6 mice (age: 6–8 weeks, weight: 22–23 g) were included to evaluate potential sex‐dependent differences in therapeutic response. Mice were assigned to MCAO/R or sham operation, and received TPNbT‐CHFn NPs or TPT‐CHFn NPs treatment. The animals were group housed in a 12‐h light/12‐h dark cycle (light between 06:00 and 18:00) in a temperature‐controlled room (22°C) with free access to water and food. Mice were randomly assigned to the control or the experimental groups. All animal experiments were conducted in accordance with the Guidelines for the Care and Use of Laboratory Animals and were approved by the Institutional Animal Care and Use Committee (IACUC) of Huazhong Agricultural University (HZAUMO‐2024‐0311).

## 7 Method Details

### 7.1 Construction and Screening of a Fully Synthetic Nanobody Library

A fully synthetic nanobody DNA library (provided by Dr. Shaoxiang Luo, Wuhan Proligand Biotechnology Co., Ltd) was constructed using trinucleotide‐directed mutagenesis based on the Lama glama IGHV1S1‐GHV1S1S5 scaffold [59]. Diversity was introduced via a two‐step overlap extension PCR (OE‐PCR). The library was cloned into the linearized pNACP vector and integrated into the yeast surface display system through in vivo homologous recombination. In this platform, nanobody sequences are fused to the C‐terminus of Aga2, which forms disulfide bonds with Aga1, thereby enabling display on the yeast cell wall. For biopanning, 150 µg of G3‐I peptide conjugated to BSA (G3‐I‐BSA) was immobilized to 6 mL of carboxyl magnetic beads (10 mg/mL, 2 µm, Beaver), and incubated with 2.5 × 10^10^ induced yeast cells. Two rounds of magnetic enrichment were performed using magnetic columns, followed by three rounds of fluorescence‐activated cell sorting (FACS) with stepwise reduction (10‐fold) in antigen concentration.

### 7.2 Next‐Generation Sequencing (NGS) and Data Analysis

NGS Sample Preparation: Each type of yeast cell, sorted by flow cytometry, was cultured at 30°C for 36 h. DNA was extracted from the yeast culture using a plasmid extraction kit (TIANGEN), and OD260 values were measured using a NanoDrop 2000 (ThermoFisher). Yeast DNA was amplified for 5 cycles using forward and reverse primers, followed by 20 cycles of amplification. The resulting amplification products were separated by electrophoresis on a 1.5% agarose gel to extract bands of 300 to 500 bp. The amplification products were concentrated using an agarose gel DNA recovery kit. NGS sequencing libraries were constructed by ligating Illumina universal sequence adapters to Illumina barcodes for paired‐end sequencing. Novogene provides comprehensive services of NGS sequencing.

Raw sequencing reads were processed using MiXCR (v3.0) with a modified assembly workflow, ensuring the seamless and full‐length assembly of nanobody sequences [60]. For downstream diversity and conservation analysis, unique CDR3 nucleotide sequences were extracted from the processed dataset. Sequence logos were subsequently generated using WebLogo (v3.7.4) to visualize positional nucleotide or amino acid conservation within the hypervariable CDR3 loop.

### 7.3 GST Pull‐Down Assay

His‐ and GST‐tagged recombinant proteins were expressed in *E. coli* BL21 and purified using Ni‐NTA resin (Qiagen) and glutathione‐agarose beads (GenScript), respectively. Equal molar amounts (1 nmol) of each protein were incubated in GST pull‐down buffer (50 mm HEPES pH 7.5, 50 mm NaCl, 2 mM EDTA pH 8.0, 0.1% NP‐40, 10% glycerol) at 4 °C for 2 h. GST‐tagged complexes were captured with glutathione beads for 30 min, washed three times, eluted in 2× SDS buffer, and subjected to Western blotting.

### 7.4 RNA Extraction

Tissue or cell samples were lysed in 1 mL TRIzol reagent (Invitrogen) and incubated at room temperature for 10 min. After centrifugation (12 000 rpm, 10 min, 4 °C), the supernatant was transferred and mixed with 200 µL chloroform. Following phase separation (12 000 rpm, 15 min), RNA was precipitated with isopropanol, washed with 75% ethanol, air‐dried, and resuspended in 35 µL RNase‐free ddH_2_O.

### 7.5 Oxygen‐Glucose Deprivation and Reoxygenation (OGD/R) Model

Cells were seeded in 6‐ or 12‐well plates and cultured to 70% confluence. After triple PBS washes, the culture medium was replaced with glucose‐ and serum‐free DMEM. Cells were transferred to a hypoxia chamber (1% O_2_, 94% N_2_, 5% CO_2_) for 2 h. Subsequently, the medium was replaced with complete medium, and cells were cultured under normoxic conditions (95% air, 5% CO_2_) for 24 h to establish the OGD/R injury model [61].

### 7.6 Assembly, Modification, and Purification of Ferritin Nanoparticles

Ferritin (HFn) and SpyCatcher‐HFn (CHFn) proteins were purified by Ni‐NTA affinity chromatography and subsequently dialyzed in ferritin assembly buffer for 24 h at 4°C to facilitate self‐assembly. The assembled CHFn nanoparticles were then mixed with either Spytag‐Nb.29E9‐TPL/S (TPNbT) or Spytag‐TPL/S (TPT) proteins at a 1:2 molar ratio, followed by buffer exchange and additional dialysis for 12 h. The resulting complexes were concentrated using 30 kDa molecular weight cutoff ultrafiltration devices.

Purification was achieved through sucrose density gradient ultracentrifugation using a 10%–50% discontinuous sucrose gradient prepared in ferritin assembly buffer. Gradients were allowed to equilibrate for 12 h at 4°C prior to sample loading. Samples were loaded and centrifuged at 38 000 rpm for 4 h at 4°C. Target fractions were collected based on density profiles, verified by SDS‐PAGE, and dialyzed against assembly buffer followed by PBS to remove sucrose. The final products (HFn, CHFn, TPT‐CHFn, and TPNbT‐CHFn NPs) were concentrated by ultrafiltration and stored at ‐80°C to minimize freeze‐thaw cycles.

For fluorescent labeling, Cy5.5‐HFn and FITC‐HFn nanoparticles were prepared by conjugating 50 nm HFn nanoparticles with 100 nm Cy5.5‐NHS ester (MedChemExpress) or FITC‐NHS ester (Yeasen Biotechnology) in PBS buffer at 4°C for 12 h with protection from light. Unreacted dyes were removed using PD MiniTrap G‐25 desalting columns (GE Healthcare). The dye‐to‐protein ratios were determined to be 10:1 for Cy5.5‐HFn and 1:1 for FITC‐HFn by UV‐vis spectroscopy (NanoDrop 2000, Thermo Fisher), with protein concentrations quantified by BCA assay (Beyotime Biotechnology).

Nanoparticle quality was assessed by transmission electron microscopy for morphological evaluation and dynamic light scattering for size distribution analysis. All purified samples were aliquoted and stored at ‐80°C until use to maintain stability.

### 7.7 Transmission Electron Microscopy (TEM)

Purified HFn nanoparticles were adsorbed onto carbon‐coated copper grids for 1 min, followed by negative staining with 1% phosphotungstic acid for 30 s. After removing excess stain, the grids were air‐dried at room temperature and imaged using a transmission electron microscope (Hitachi, 100 kV) over several randomly selected fields. All procedures were performed in a laminar flow hood to avoid contamination.

### 7.8 Dynamic Light Scattering (DLS)

HFn nanoparticles were filtered (0.22 µm) or centrifuged (12 000 rpm, 15 min, 4 °C), and 10 µL was transferred to a plastic cuvette for measurement using a DynaPro NanoStar (Wyatt Technology). Parameters: 25 °C, water as solvent, multi‐angle detection mode, three cycles, one measurement per cycle. Samples were recovered post‐analysis.

### 7.9 Co‐Immunoprecipitation (Co‐IP)

Cells were lysed in non‐denaturing lysis buffer (without DTT) supplemented with 0.5% protease inhibitor cocktail. After 30 min rotation at 4°C, lysates were sonicated (12% power, 2 s on, 5 s off, five cycles) and centrifuged (12 000 rpm, 10 min, 4°C). Supernatants were incubated with 1 µg of primary antibody overnight at 4°C, followed by 4 h incubation with protein A‐conjugated Sepharose beads. Beads were washed four times with wash buffer to remove nonspecific interactions. Bound proteins were eluted with 2× SDS loading buffer at 98°C for 10 min and analyzed by SDS‐PAGE and immunoblotting.

### 7.10 Hematoxylin and Eosin (H&E) Staining

For toxicological assessment, major organs (heart, liver, spleen, lung, kidney) were harvested at days 0, 4, 7, and 14 post‐administration of TPNbT‐CHFn NPs. Tissues were fixed in 10% neutral‐buffered formalin, paraffin‐embedded, sectioned at 4 µm, and stained with H&E. Sections were evaluated for histopathological changes using a BX53 light microscope (Olympus).

### 7.11 MCAO/R Model Establishment

C57BL/6 mice (6–8 weeks old, 22–23 g) were subjected to MCAO followed by reperfusion (MCAO/R), with sham‐operated animals serving as controls [62]. Briefly, mice were anesthetized with isoflurane (30% O_2_/70% N_2_O), and a midline neck incision was made to expose the right common carotid artery (CCA) bifurcation. The external carotid artery (ECA) was ligated, and a silicone‐coated nylon monofilament (rounded tip) was introduced through the ECA stump into the internal carotid artery to occlude the MCA origin (insertion depth: 10 ± 0.5 mm). After 60 min of occlusion, the filament was withdrawn to initiate reperfusion. Mice of the sham‐operated group were performed the same as the MCAO/R procedure without filament insertion. Core body temperature was maintained at 36.5–37.5°C throughout surgery using a heating pad, and postoperative analgesia (meloxicam) was administered. Experimental groups: MCAO/R mice; Control group: Sham‐operated mice.

### 7.12 Cellular Uptake of Ferritin Nanoparticles

FITC‐labeled HFn, CHFn, and TPNbT‐CHFn NPs (0.4 µm) were used to evaluate uptake efficiency in bEnd.3 (brain microvascular endothelial cells), HT22 (neurons), and BV2 (microglia). Cells were seeded in 12‐well plates at 4 × 10^5^ cells/well and allowed to adhere overnight (37°C, 5% CO_2_). After removing culture medium, NPs in complete medium were added for 1 h incubation. Following uptake termination with ice‐cold PBS washes, FITC fluorescence was detected by spinning‐disk confocal microscopy (NIKON, λex/λem = 488/519 nm). For flow cytometry (Beckman Coulter), trypsinized cells were filtered through 40 µm mesh and analyzed with FlowJo V10.8 to determine uptake‐positive cell percentages.

### 7.13 In Vitro Blood‐Brain Barrier Transcytosis Assay

Model Establishment: Transwell inserts (0.4 µm, Corning) were pre‐coated with Matrigel (50 µL/cm^2^, Corning) on ice and polymerized at 37°C for 30 min. bEnd.3 cells (6 × 10^4^ cells/cm^2^) were seeded in the upper chamber, with HT22 or BV2 cells (same density) optionally cultured in the lower chamber. After 48–72 h co‐culture, TEER values reached 100 Ω·cm^2^ (Millicell ERS‐2).

Delivery Assessment: FITC‐labeled NPs (50 µg, HFn/CHFn/TPNbT‐CHFn) were added to the upper chamber. Basolateral medium was collected at 30/60/120 min for fluorescence (495 nm) and protein (280 nm) measurement (BioTek microplate reader). Transcytosis efficiency was calculated as fluorescence/protein ratio. For co‐culture groups, lower chamber cells were trypsinized at 120 min for flow cytometric quantification of cellular uptake. Experiments were triplicated.

### 7.14 Brain Distribution Assay

C57BL/6 mice (6–8 weeks old) were depilated 24 h before imaging to minimize autofluorescence. Under isoflurane anesthesia, the head was further shaved and treated with depilatory cream (Veet), followed by cleaning with alcohol. Mice were injected intravenously via the tail vein with Cy5.5‐labeled NPs (10 mg/kg, 200 µL). In vivo fluorescence imaging was performed at 0, 3, 6, and 24 h post‐injection using a pre‐warmed (37°C) IVIS Spectrum CT system (λex/λem = 675/700 nm). After the final imaging session, the mice were euthanized, and the major organs were harvested for ex vivo imaging to evaluate the biodistribution of Cy5.5‐NPs. Cy5.5‐specific signals were extracted using Living Image software (version 4.4) after background subtraction.

### 7.15 Behavioral and mNSS Tests

To evaluate motor function following unilateral brain interventions, we conducted a series of behavioral tests including beam walking, horizontal ladder, and rotarod assays. All assessments were performed preoperatively and at multiple time points over a 6‐week postoperative period, with particular attention to right limb performance contralateral to the left hemispheric interventions.

For the beam walking test, mice were first acclimated in a dark environment for 2 h. Animals were then placed at one end of a 1‐meter long, 4‐cm wide square wooden beam and allowed to spontaneously traverse to a dark box at the opposite end. Two experimenters positioned on either side of the beam recorded the number of right forelimb slips during a maximum 5‐min observation period, with performance scored on a 10‐point scale (10 minus number of slips). Each mouse completed three trials, with the average score used for analysis.

The horizontal ladder test utilized an apparatus consisting of two side panels (70 × 15 cm) with irregularly spaced rungs (5 cm intervals). Following 2 h of dark adaptation, mice were placed at the starting point and their traversal time (excluding pauses) was recorded up to a maximum of 300 s. The number of right limb slips was counted and converted to a 10‐point score (10 minus slips). Only animals demonstrating baseline traversal capability (completing the task within 180 s during training sessions) were included in formal testing.

Motor coordination was assessed using a rotarod set to constant rotation (30 rpm). After 2–3 acclimation trials, mice underwent three formal test trials separated by 30‐min intervals. The primary outcome measure was latency to fall (maximum 300 s), with the rotational speed at time of fall recorded as a secondary parameter. The apparatus featured physically separated lanes to prevent interference between simultaneously tested animals.

Neurological deficit was evaluated using the modified neurological severity score (mNSS), a composite measure of motor, balance, and reflex function, as described previously (Table S2) [63]. Scores range from 0 (normal function) to 10 (maximal impairment). On each testing day, three measurements were obtained per animal.

All behavioral scoring was performed by experimenters blinded to treatment conditions, with particular focus on right limb performance to assess deficits resulting from left hemispheric interventions. Standardized pretest handling and dark adaptation procedures were implemented across all assessments to minimize stress‐related variability. Equipment specifications including beam dimensions (1 m × 4 cm), ladder rung spacing (5 cm irregular), and rotarod parameters (30 rpm constant speed) were rigorously maintained throughout the study to ensure consistency.

### 7.16 Western Blotting

The total protein was obtained by RIPA lysis buffer (Beyotime Biotechnology, China) on ice and the concentration was examined by BCA Protein Assay Kit (Beyotime Biotechnology, China). Protein samples were subjected to SDS‐PAGE. Separated proteins were transferred to methanol‐activated PVDF membranes using a wet transfer system (200 mA, ice‐cooled), with the gel oriented toward the negative electrode. Membranes were blocked for 1 h, then incubated with primary antibodies at 4°C overnight. After three 15‐min TBST washes, HRP‐conjugated secondary antibodies were applied for 4 h at 4°C. Following repeated washes, protein bands were visualized using ECL substrate and quantified via chemiluminescence imaging (ChemiScope 6000 Exp).

### 7.17 Immunofluorescence Staining

For cultured cells: Cells grown on glass coverslips were fixed with 4% paraformaldehyde (30 min, RT), permeabilized with 0.25% Triton X‐100 (10 min), and blocked with 5% BSA (1 h, RT). Primary antibodies against His (ABclonal, 1:100), Iba1 (Abcam, 1:100), MAP2 (Proteintech, 1:500), NeuN (Abcam, 1:100), Active Caspase3 (BD Biosciences, 1:500), CD16/32 (BD Biosciences, 1:200), CD206 (Cell Signaling, 1:500), GFAP (CST, 1:1000), Syn1 (Proteintech, 1:500) and CD31 (Abcam, 1:100) were applied overnight at 4°C. After washing, cells were incubated with appropriate secondary antibodies (1 h, RT, dark) and counterstained with DAPI. Coverslips were mounted and imaged using a spinning‐disk confocal microscope (Andor Technology).

For brain tissue sections: Mice were transcardially perfused with normal saline for 5 min, followed by 4% paraformaldehyde (PFA) for 5 min. Brains were post‐fixed overnight (4°C), cryoprotected in a graded sucrose series (10%, 20%, and 30% in PBS) at 4°C until they sank, and embedded in OCT compound. Coronal sections (25 µm) were cut using a cryostat (Leica) and processed for immunofluorescence using the same staining protocol as for cultured cells. For each animal, 3–5 consecutive coronal sections (25 µm) from ‐0.1 to +0.3 mm relative to bregma were analyzed. In the ipsilateral cortex and striatum, three non‐overlapping fields per section were randomly selected for positive cell counting or mean fluorescence intensity analysis. Values per animal were averaged, giving 6 independent data points per condition.

### 7.18 TTC Staining

Brain sections (0.2 mm thickness) were incubated in pre‐warmed 2% TTC solution (37°C, 20 min with one flip) to assess ischemic infarction. After fixation with 4% PFA (4°C, 12–24 h), infarct areas (unstained white regions) were quantified using ImageJ and normalized to contralateral hemisphere area.

### 7.19 Nissl Staining

Brain sections were stained with methylene blue solution (65°C, 10 min), differentiated with Nissl differentiation solution (65°C, 3 min), and counterstained with ammonium molybdate (65°C, 5 min). After ethanol dehydration and xylene clearing, sections were mounted with neutral resin for neuronal architecture analysis. An investigator blinded to groups used ImageJ to delineate the lesioned area and count healthy neurons per unit area (mm^2^).

### 7.20 Quantification and Statistical Analysis

All data are presented as mean ± standard error of the mean (SEM). Sample sizes (*n*) refer to biological replicates (independent animals or independent cell preparations, as specified in figure legends). For histological and imaging analyses, 3–5 serial coronal brain sections (25 µm thickness) spanning from ‐0.1 mm to +0.3 mm relative to bregma were analyzed per animal, covering both the ischemic core and penumbra (primarily cortex and striatum). Regions of interest (ROIs) were delineated according to standardized anatomical landmarks under a stereotaxic atlas‐guided template, as illustrated in Figure 5F. All image acquisition and quantitative analyses were performed by a single experimenter blinded to the experimental group allocation. Behavioral scoring was likewise conducted in a blinded manner, as detailed under “Behavioral and mNSS tests.”

Statistical analyses were performed using GraphPad Prism v9. For comparisons between two independent groups, unpaired two‑tailed Student's *t*‑test was used. For comparisons among three or more independent groups, one‑way ANOVA was applied, followed by Tukey's multiple comparisons test. When repeated measurements over time were analyzed (e.g., neurobehavioral assessments on days 1, 3, and 7 post‑surgery; in vivo fluorescence imaging at 0, 3, 6, and 24 h; in vitro transcytosis time‑course), two‑way repeated‑measures ANOVA was used with Greenhouse‐Geisser correction when sphericity was violated. Post‑hoc comparisons for two‑way ANOVA were performed using Dunnett's or Sidak's multiple comparisons test, as specified in the respective figure legends. Statistical significance was annotated as ns (not significant), ^*^ *p* < 0.05, ^**^ *p* < 0.01, and ^***^ *p* < 0.001.

Sample sizes were determined based on established conventions in published MCAO/R literature. Because a prospective a priori power analysis was not conducted, we performed a retrospective sensitivity power analysis to assess the robustness of our findings. Using G^*^Power 3.1, we computed the minimum detectable effect size (Cohen's *f*) that could be detected with our actual sample sizes (*n* = 3–7 per group) at α = 0.05 and power = 0.80. For experiments with *n* = 6–7 (behavior, immunofluorescence, TTC, Nissl, mNSS, ELISA), the design was sensitive to detect large effects (*f* ≥ 0.48), and all significant outcomes exceeded this threshold. For experiments with *n* = 3 per group (MDA, SOD, brain volume, MRI), sensitivity analysis indicated that only very large effects (*f* ≥ 1.05) were detectable with 80% power; the observed effect sizes in these experiments (Cohen's *f* = 3.89–7.00) all markedly surpassed this conservative threshold, confirming that our sample sizes were sufficient to detect the effects of interest. Calculated Cohen's *f* values for each primary endpoint are provided in Table S3.

### 7.21 Manuscript Preparation

During manuscript drafting, AI tools (ChatGPT and DeepSeek) were used solely for text refinement (grammar, syntax, and readability). All AI‐generated content was rigorously verified, edited, and approved by the authors to ensure scientific accuracy.

## Author Contributions

M.Z. and Z.D. conceived the project. M.Z., Z.D., L.L. and M.L. designed the project. L.L., M.L., L.S., Y.Y., Y.W., Z.Y., A.W., P.Z., S.L., J.C., J.Q., Z.A. and Z.Y. performed the experimental work. M.Z., Z.D., L.L. and M.L. analyzed the results and wrote the manuscript. All authors edited and approved the manuscript.

## Funding

The National Natural Science Foundation of China grants (No. 31970682, No. 81770314 and No. 32571174), the Fundamental Research Funds for the Central Universities (No. 2662023PY019) and the Knowledge Innovation Program of Wuhan‐Basic Research (No. 2022020801010511 and No. 2023020201010104).

## Ethics Statement

We thank the Institutional Animal Care and Use Committee (IACUC) of Huazhong Agricultural University for Innovation (HZAUMO‐2024‐0311) for supporting the mouse studies and the Instruments Sharing Plat‐form of College of Biomedicine and Health, College of Life science and Technology, Huazhong Agricultural University.

## Conflicts of Interest

The authors declare no conflicts of interest.

## Supporting information




**Supporting File**: advs76004‐sup‐0001‐SuppMat.docx.

## Data Availability

Protein profile data have been deposited at PRIDE and are publicly available as of the date of publication. Accession numbers are listed in the key resources table. All data reported in this paper will be shared by the lead contact upon request. Reviewer access details Log in to the PRIDE website using the following details: Project accession: PXD067942; Token: jsi7fs6plsrp. Alternatively, reviewer can access the dataset by logging in to the PRIDE website using the following account details: Username: reviewer_pxd067942@ebi.ac.uk; Password: BZNOH6iCCe51. Any additional information required to reanalyze the data reported in this paper is available from the lead contact upon request. Further information and requests for resources and reagents should be directed to and will be fulfilled by the lead contact, **Min Zhang** (minzhang@mail.hzau.edu.cn). All unique materials generated in this study are available upon request. All requests for resources and reagents should be directed to the lead contact.
