# Supplementary material for: A Brain‐Penetrant Nanobody Reveals GSK3β‐Driven Proline‐Directed Phosphorylation as a Master Regulator of Ischemic Neurodegeneration
Source: Adv Sci (Weinh). 2026 Jun 9:e76004. Online ahead of print. doi: 10.1002/advs.76004 (PMC13337028; doi:10.1002/advs.76004)
Supplement: Supplementary file 1 — Supporting File: advs76004‐sup‐0001‐SuppMat.docx. [file ADVS-9999-e76004-s001.docx]

**SUPPLEMENTAL INFORMATION**

**A Brain-Penetrant Nanobody Reveals GSK3β-Driven Proline-Directed Phosphorylation as a Master Regulator of Ischemic Neurodegeneration**

**Figures S1-S7 and Table S1-S3**

Supporting Information

**A Brain-Penetrant Nanobody Reveals GSK3β-Driven Proline-Directed Phosphorylation as a Master Regulator of Ischemic Neurodegeneration**

*Lan Li, Muyang Li, Lei Sun, Ying Yang, Yuanshun Wu, Ziyi Yin, Anni Wang, Peiyang Zhou, Shaoxiang Luo, Jian Chen, Jun Qin, Zhibing Ai, Zilong Yuan, Zhiqiang Dong*, and Min Zhang**

**
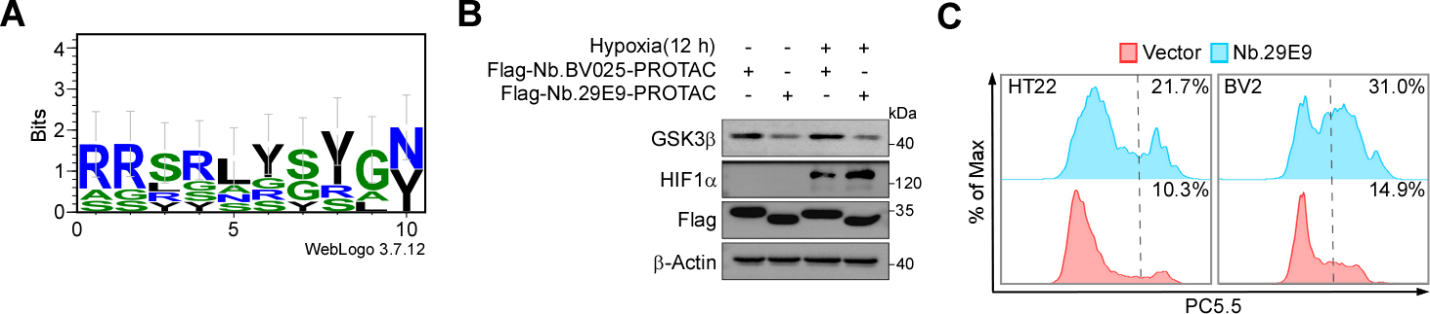
**

**Figure S1. Identification of high-affinity nanobodies targeting GSK3β, related to Figure 1.**

(A) Sequence alignment of the top 10 high-affinity nanobody clones reveals extensive diversity within the CDR3 regions.

(B) HeLa cells were transfected with FLAG-tagged Nb.29E9-PROTAC or Nb.BV025-PROTAC for 36 h and subjected to 12 h hypoxia. Western blot analysis confirms that Nb.29E9-PROTAC mediates degradation of endogenous GSK3β under both normoxic and hypoxic conditions.

(C) Flow cytometric analysis of PI staining in HT22 neurons and BV2 microglia after overexpression of Vector or Nb.29E9.


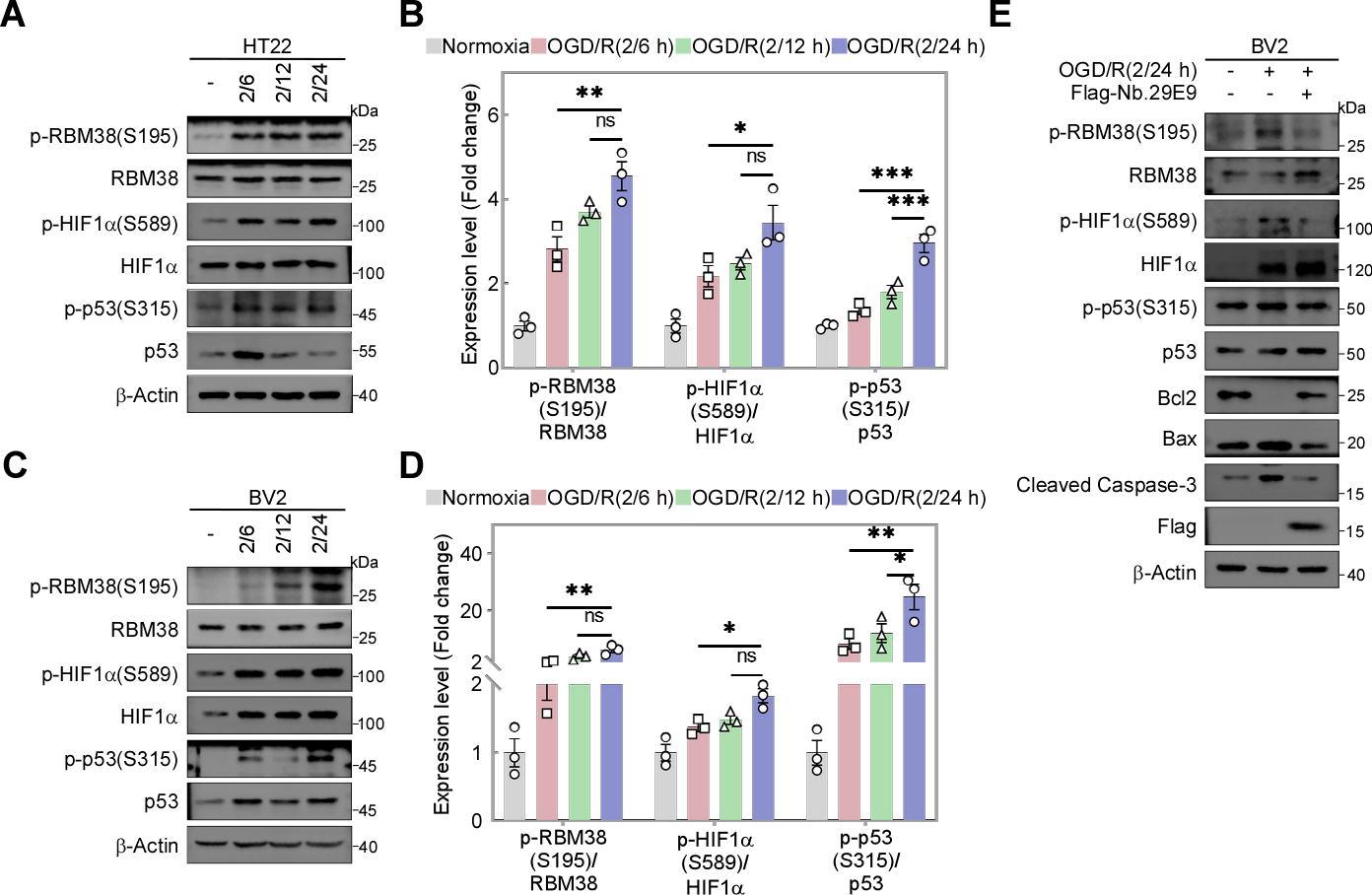


**Figure S2. Activation of GSK3β S/T-P kinase signaling under OGD/R, related to Figure 2.**

(A) Western blot analysis was performed to assess the phosphorylation levels of RBM38 (Ser195), HIF1α (Ser589), and p53 (Ser315) in HT22 cells.

(B) Quantification of phosphorylation levels of RBM38 (Ser195), HIF1α (Ser589), and p53 (Ser315). Data are mean ± SEM (*n* = 3 per group). *p* values versus OGD/R (2/12 h) group: p-RBM38, *p =* 0.0850; p-HIF1α, *p =* 0.0686; p-p53, *p* < 0.001; *p* values versus OGD/R (2/6 h) group: p-RBM38, *p =* 0.0022; p-HIF1α, *p =* 0.0207; p-p53, *p* < 0.001; one-way ANOVA with Tukey's multiple comparisons test.

(C) Western blot analysis was performed to assess the phosphorylation levels of RBM38 (Ser195), HIF1α (Ser589), and p53 (Ser315) in BV2 cells.

(D) Quantification of phosphorylation levels of RBM38 (Ser195), HIF1α (Ser589), and p53 (Ser315). Data are mean ± SEM (*n* = 3 per group). *p* values versus OGD/R (2/12 h) group: p-RBM38, *p =* 0.0678; p-HIF1α, *p =* 0.0589; p-p53, *p* = 0.0348; *p* values versus OGD/R (2/6 h) group: p-RBM38, *p =* 0.0025; p-HIF1α, *p =* 0.0181; p-p53, *p* = 0.0091; one-way ANOVA with Tukey's multiple comparisons test.

(E) Western blot analysis of apoptosis-related and phosphorylated proteins in BV2 cells following OGD/R. ns = not signiﬁcant, **p* < 0.05, ***p* < 0.01, ****p* < 0.001.

**
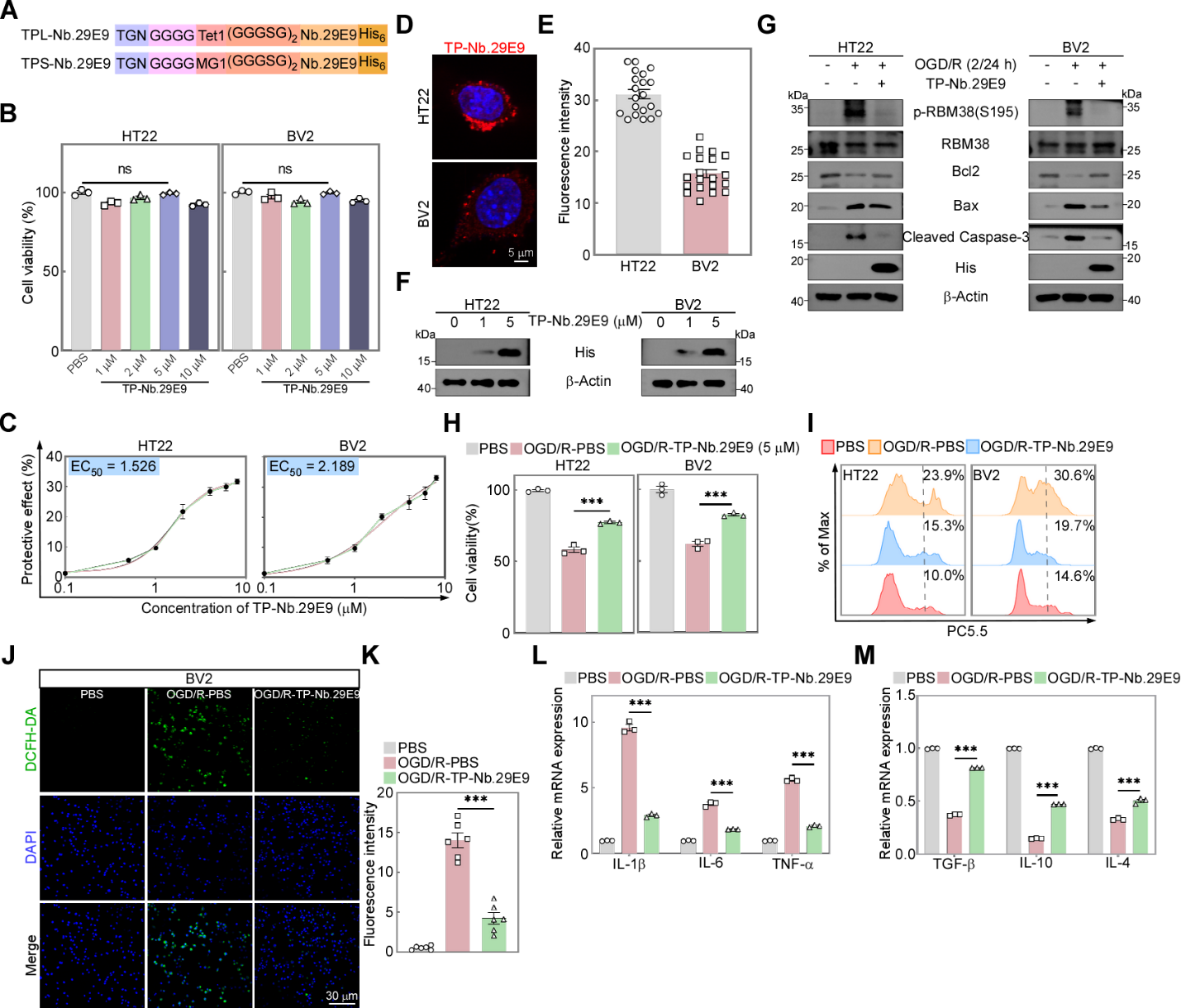
**

**Figure S3. TP-Nb.29E9 alleviates OGD/R-induced apoptosis, oxidative stress, and inflammation, related to Figure 3.**

(A) Schematic of the TP-Nb.29E9 fusion construct.

(B) CCK8 assays evaluating cell viability of HT22 and BV2 after 24 h incubation with TP-Nb.29E9-His6 at 1, 2, 5, or 10 μM (*n* = 3).

(C) HT22 and BV2 cells were subjected to oxygen‑glucose deprivation for 2 h followed by 24 h of reperfusion (OGD/R) in the presence of TP-Nb.29E9 at the indicated concentrations (0.1, 0.5, 1, 2, 4, 6, and 8 µM) or an equivalent volume of PBS vehicle. Cell viability was assessed by CCK8 assay and normalized to the normoxic control group. Data are mean ± SEM (*n* = 3 per group). The concentration-response curve was fitted using a four-parameter logistic regression model (variable slope) in GraphPad Prism. HT22 cells: EC₅₀ = 1.526 µM. BV2 cells: EC₅₀ = 2.189 µM.

(D-E) Fluorescence microscopy confirming cellular uptake of 5 μM TP-Nb.29E9-His6 after 2 h incubation (scale bar = 10 μm). For quantification of single-cell fluorescence intensity, 20 cells from 3 biological replicate were analyzed. Data are mean ± SEM.

(F) Western blot analysis of cells incubated with 1 or 5 μM TP-Nb.29E9-His6 for 2 h, followed by 10 h culture in fresh medium.

(G) HT22 and BV2 cells were subjected to 2 h OGD, followed by 24 h reoxygenation in the presence of 5 μM TP-Nb.29E9. Western blot analysis shows decreased RBM38-S195 phosphorylation and modulation of apoptosis markers (Bcl2, Bax, Cleaved Caspase-3).

(H) Viability of HT22 and BV2 cells following OGD/R exposure. Cells were subjected to OGD/R in the presence of TP-Nb.29E9. Data are mean ± SEM (*n* = 3 per group). *p* < 0.001 versus OGD/R-PBS group; one-way ANOVA with Tukey's multiple comparisons test.

(I) Flow cytometric analysis of cell death assessed by propidium iodide (PI) staining following OGD/R exposure.

(J) Representative immunofluorescence images showing ROS accumulation (green) in BV2 cells (scale bar = 30 μm).

(K) Quantification of ROS intensity in BV2 cells. Data are presented as mean ± SEM (*n* = 6 per group). Statistical significance was assessed using one-way ANOVA with Tukey’s multiple comparisons test; *p* < 0.001 versus OGD/R-PBS group.

(L-M) qPCR analysis of pro- and anti-inflammatory cytokine expression. Data are mean ± SEM (*n* = 3). *IL-1β*: *p* < 0.001; *IL-6*: *p* < 0.001; *TNF-α*: *p* < 0.001; *TGF-β*: *p* < 0.001; *IL-10*: *p* < 0.001; *IL-4*: *p* < 0.001; one-way ANOVA followed by Tukey's multiple comparisons test. ns = not signiﬁcant, **p* < 0.05, ***p* < 0.01, ****p* < 0.001.

**
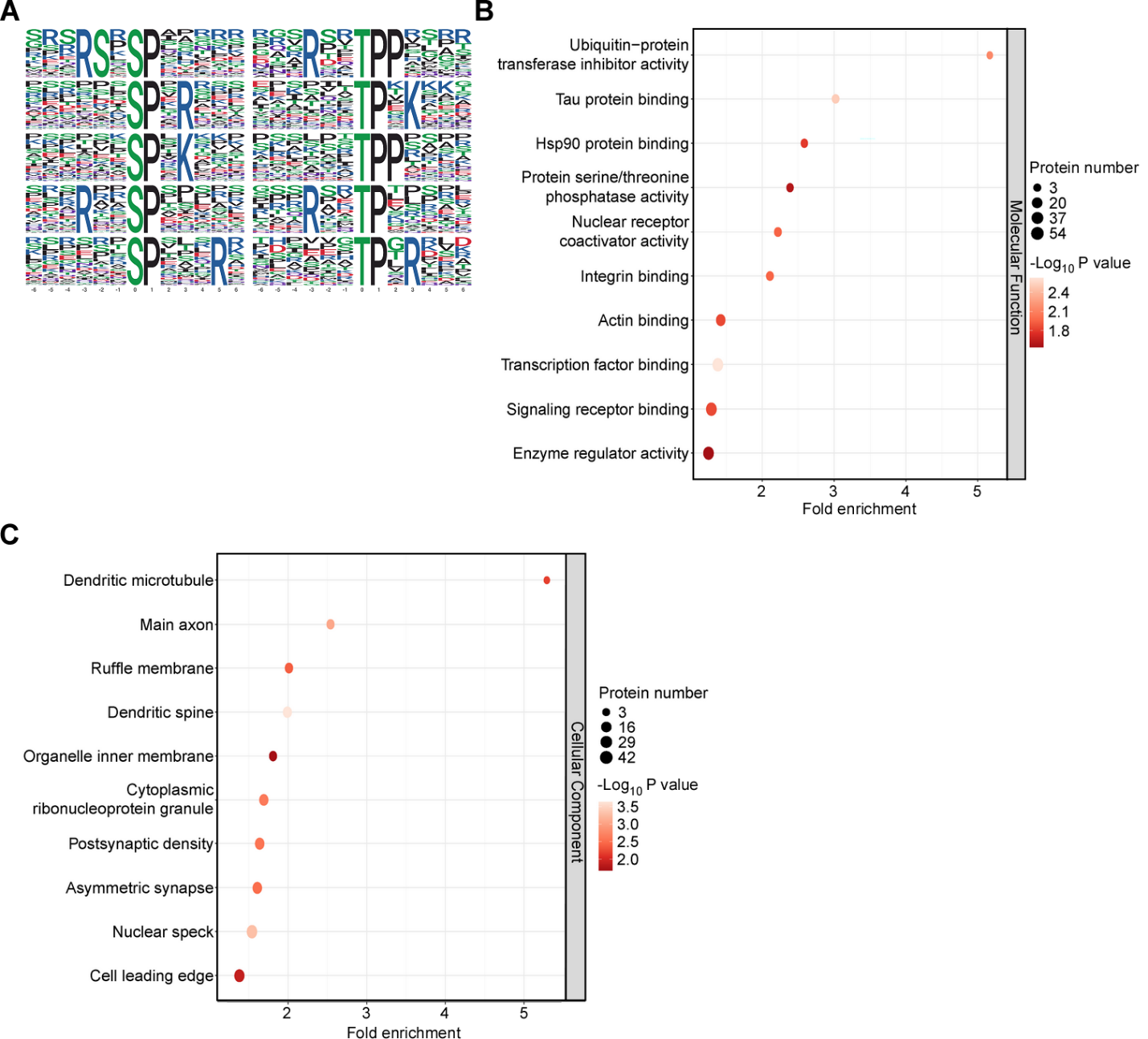
**

**Figure S4. Phosphoproteomic profiling of HT22 cells treated with TP-Nb.29E9, related to Figure 3.**

(A) Motif analysis of phosphorylation sites identifies 10 conserved sequence motifs centered on S/T residues, with letter size indicating amino acid frequency.

(B-C) Gene Ontology enrichment of differentially phosphorylated proteins, highlighting significant terms in (B) molecular function and (C) cellular component categories.

**
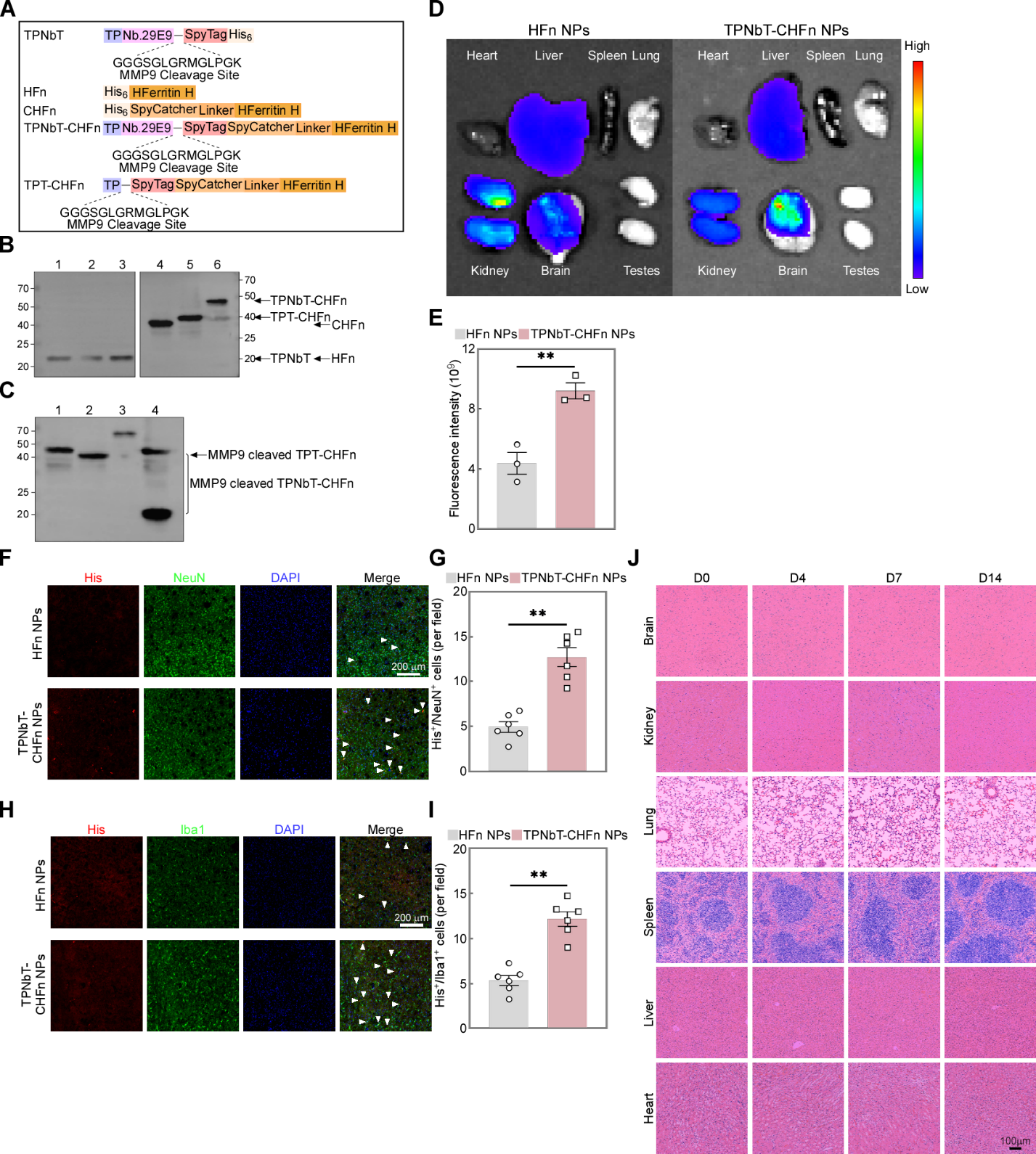
**

**Figure S5. Characterization and biosafety evaluation of engineered ferritin nanoparticles, related to Figure 4.**

(A) Schematic diagram of recombinant protein constructs.

(B) Western blot analysis of individual components including TPNbT (lanes 1-2), HFn (lane 3), CHFn (lane 4), TPT-CHFn (lane 5), and TPNbT-CHFn (lane 6).

(C) Validation of MMP-9-mediated cleavage of TPT-CHFn and TPNbT-CHFn by Western blot. Lane 1: purified TPT-CHFn; lane 2: MMP-9-digested TPT-CHFn; lane 3: purified TPNbT-CHFn; lane 4: MMP-9-digested TPNbT-CHFn.

(D) Representative *ex vivo* fluorescence images of major organs harvested 24 h post-injection. Organs shown include the brain, heart, liver, spleen, lungs, kidneys, and testes.

(E) Quantitative analysis of fluorescence efficiency in *ex vivo* brain. Data are mean ± SEM (*n* = 3 per group). *p* < 0.001 versus HFn NPs group; one-way ANOVA with Tukey's multiple comparisons test.

(F) Representative immunofluorescence images of brain sections stained for His (red) and NeuN (green). Arrows indicate His⁺/NeuN⁺ double-positive cells. Scale bar = 200 μm.

(G) Quantitative of His⁺/NeuN⁺ cell numbers per field. Data are mean ± SEM (*n* = 3 mice per group). *p* = 0.0015 versus HFn NPs group (unpaired t-test).

(H) Representative immunofluorescence images of brain sections stained for His (red) and Iba1 (green). Arrows indicate His⁺/Iba1⁺ double-positive cells. Scale bar = 200 μm.

(I) Quantitative analysis of His⁺/Iba1⁺ cell numbers per field. Data are mean ± SEM (*n* = 3 mice per group). *p* = 0.0017 versus HFn NPs group (unpaired t-test).

(J) H&E staining of major organs at day 0, 4, 7, and 14 following intravenous administration of TPNbT-CHFn NPs (50 mg/kg) confirms biocompatibility.

**
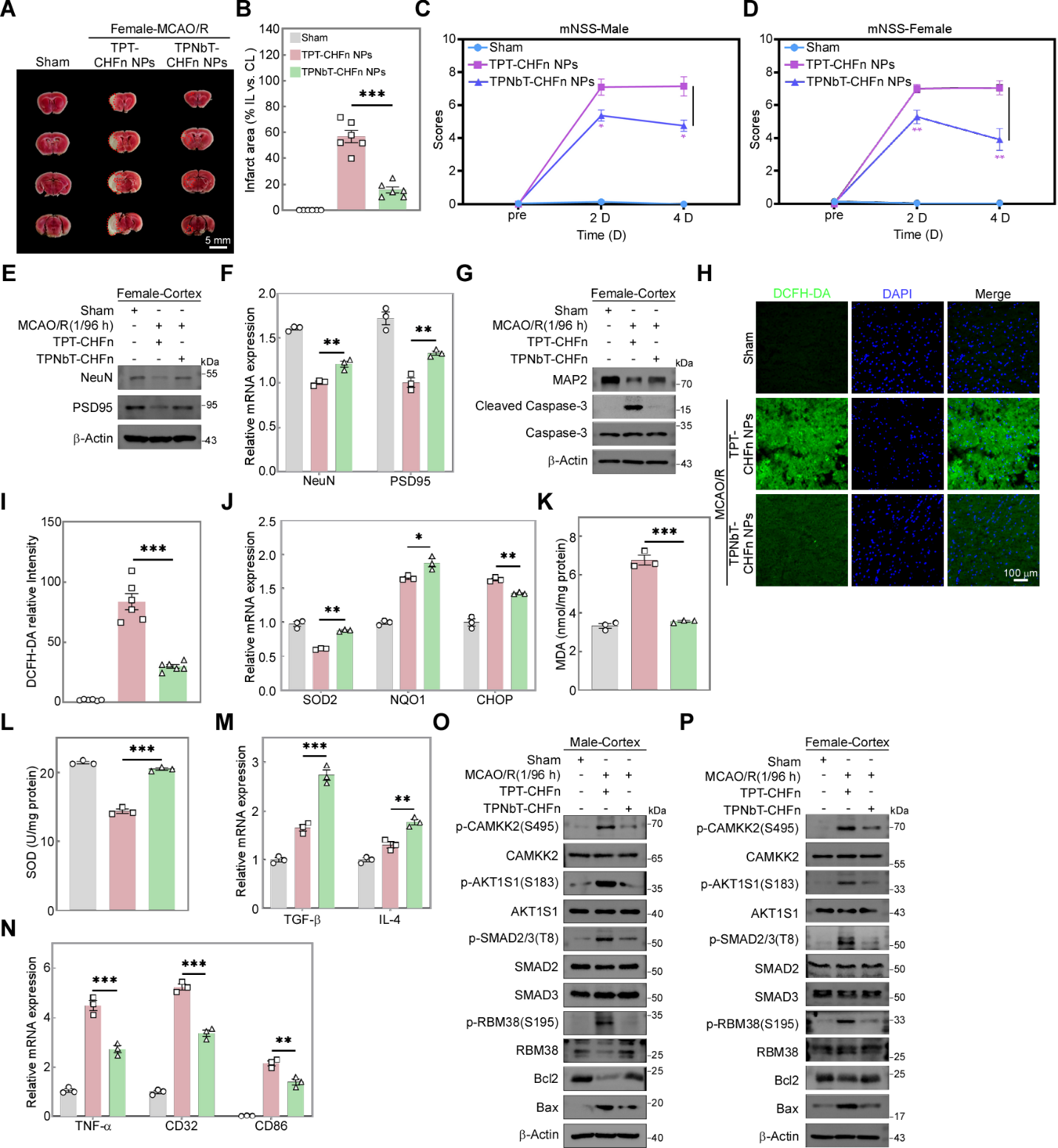
**

**Figure S6. TPNbT-CHFn NPs attenuate acute and subacute ischemic injury in MCAO/R mice, related to Figure 5.**

(A) Representative TTC-stained brain sections from female MCAO/R mice injected intravenously with TPT-CHFn NPs or TPNbT-CHFn NPs immediately upon reperfusion (0 h) and again at 48 h post-reperfusion, alongside sham-operated controls.

(B) Quantitative analysis of infarct volume in brain sections stained with TTC at 96 h post-reperfusion. Data are mean ± SEM (*n* = 6 per group). *p* < 0.001 versus TPT-CHFn NPs group; one-way ANOVA with Tukey’s multiple comparisons test.

(C-D) mNSS at baseline (pre-surgery), and at 2 and 4 days after MCAO/R. Mice treated with TPNbT-CHFn NPs showed significantly better neurological outcomes over the 4-day assessment period compared with the MCAO/R + TPT-CHFn NPs group. Data are mean ± SEM (*n* = 7 mice per group). Male: *p* < 0.001; Female: *p* < 0.001, versus MCAO/R+ TPT-CHFn NPs group; two-way repeated-measures ANOVA followed by post hoc multiple-comparisons tests (Greenhouse-Geisser corrected).

(E) Western blot analysis of NeuN and PSD95 protein levels in the ischemic cortex of female mice subjected to MCAO and treated with TPT-CHFn NPs or TPNbT-CHFn NPs, alongside sham-operated controls.

(F) qPCR analysis of *NeuN* and *PSD95* mRNA levels in ischemic cortex. Data are presented as mean ± SEM (*n* = 3 per group). *NeuN*: *p* = 0.0015; *PSD95*: *p* = 0.0079, versus MCAO/R+ TPT-CHFn NPs group; one-way ANOVA with Tukey's multiple comparisons test.

(G) Western blot analysis of MAP2, Cleaved Caspase-3 and Caspase-3 protein levels in the ischemic cortex of female mice subjected to MCAO and treated with TPT-CHFn NPs or TPNbT-CHFn NPs, alongside sham-operated controls.

(H) Representative immunofluorescence images of DCFH-DA (green) fluorescence indicating ROS levels in brain sections (scale bar = 100 μm).

(I) Quantitative analysis of ROS (green) fluorescence efficiency. Data are mean ± SEM (*n* = 6 per group). *p* < 0.001 versus TPT-CHFn NPs group; one-way ANOVA with Tukey's multiple comparisons test.

(J) qPCR analysis of *SOD2*, *NQO1*, and *CHOP* mRNA levels in ischemic cortex. Data are mean ± SEM (*n* = 3 per group). *SOD2*: *p* < 0.001; *NQO1*: *p* = 0.0092; *CHOP*: *p* = 0.0065, versus the MCAO/R+ TPT-CHFn NPs group; one-way ANOVA with Tukey's multiple comparisons test.

(K-L) Biochemical assays show reduced MDA levels and increased SOD activity, indicating attenuated oxidative damage (*n =* 3 mice per group). Data are mean ± SEM. *p* < 0.001 versus TPT-CHFn NPs group; one-way ANOVA with Tukey's multiple comparisons test.

(M-N) qPCR analysis of anti-inflammatory cytokines (*TGF-β*, *IL-4*) and M1 markers (*TNF-α, CD32*, *CD86*) expression. Data are mean ± SEM (*n* = 3). *TGF-β*: *p* < 0.001; *IL-4*: *p* = 0.0013; *TNF-α*: *p* < 0.001; *CD32*: *p* < 0.001; *CD86*: *p* = 0.0015, versus the MCAO/R+ TPT-CHFn NPs group; one-way ANOVA with Tukey's multiple comparisons test.

(O-P) Western blot analysis of apoptosis-related and phosphorylated proteins in the ischemic cortex of male and female mice subjected to MCAO and treated with TPT-CHFn NPs or TPNbT-CHFn NPs, alongside sham-operated controls. ns = not signiﬁcant, **p* < 0.05, ***p* < 0.01, ****p* < 0.001.


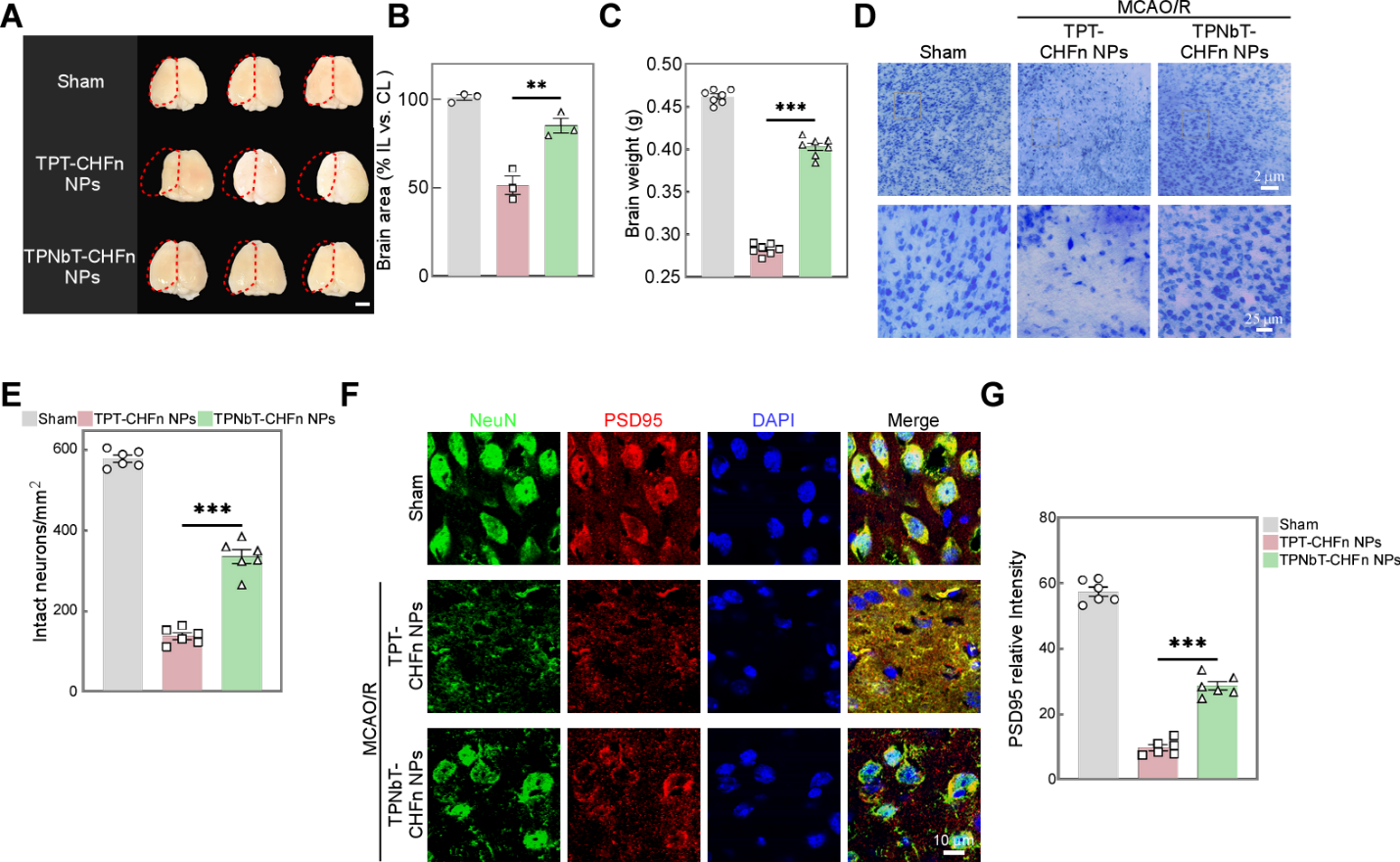


**Figure S7. TPNbT-CHFn NPs reduce chronic brain atrophy and preserve neuronal integrity at 6 weeks after MCAO/R, related to Figure 6.**

(A-B) Representative images of cerebral atrophy at 6 weeks post-treatment. Data are mean ± SEM (*n* = 3). The ischemic hemispheres are marked by dotted lines (scale bar = 2 mm). *p* = 0.0013 versus TPT-CHFn NPs group; one-way ANOVA followed by Tukey's multiple comparisons test.

(C) Quantification of brain weight at week 6. Data are mean ± SEM (*n* = 7). *p* < 0.001 versus TPT-CHFn NPs group; one-way ANOVA followed by Tukey's multiple comparisons test.

(D-E) Representative images of Nissl staining reveals preserved neuronal density. Data are mean ± SEM (*n* = 6). *p* < 0.001 versus TPT-CHFn NPs group; one-way ANOVA followed by Tukey's multiple comparisons test.

(F-G) Representative images of immunofluorescence staining of NeuN (green) and PSD95 (red) at 6 weeks post-treatment demonstrates increased synaptic marker expression (scale bar = 100 μm). Data are mean ± SEM (*n* = 6). *p* < 0.001 versus TPT-CHFn NPs group; one-way ANOVA followed by Tukey's multiple comparisons test. ns = not signiﬁcant, **p* < 0.05, ***p* < 0.01, ****p* < 0.001.

## Table S1. qRT-PCR primer used in this study, related to Figures 2H, 5I, 5J, S3H and S6B.

| Gene name | Primer name | Primer sequence (5’-3’) |
| --- | --- | --- |
| *PSD95*  (mouse) | Forward | TCAGACGGTCACGATCATCGCT |
|  | Reverse | GTTGCTTCGCAGAGATGCAGTC |
| *RBFOX3*/NeuN  (mouse) | Forward | CACCACTCTCTTGTCCGTTTGC |
|  | Reverse | GGCTGAGCATATCTGTAAGCTGC |
| *CHOP*  (mouse) | Forward | GGAAACAGAGTGGTCATTCCC |
|  | Reverse | CTGCTTGAGCCGTTCATTCTC |
| *NQO1*  (mouse) | Forward | GAAGAGCACTGATCGTACTGGC |
|  | Reverse | GGATACTGAAAGTTCGCAGGG |
| *IL1*  (mouse) | Forward | GCAACTGTTCCTGAACTCAACT |
|  | Reverse | ATCTTTTGGGGTCCGTCAACT |
| *IL6*  (mouse) | Forward | TAGTCCTTCCTACCCCAATTTCC |
|  | Reverse | TTGGTCCTTAGCCACTCCTTC |
| *TNFα*  (mouse) | Forward | TCTCAGCCTCTTCTCATTCCTGCT |
|  | Reverse | AGAACTGATGAGAGGGAGGCCATT |
| *TGFβ*  (mouse) | Forward | GGTCTCAACCCCCAGCTAGT |
|  | Reverse | GCCGATGATCTCTCTCAAGTGAT |
| *IL10*  (mouse) | Forward | GCTCTTACTGACTGGCATGAG |
|  | Reverse | CGCAGCTCTAGGAGCATGTG |
| IL4  (mouse) | Forward | TCTGCATTGCACTTATGCTGA |
|  | Reverse | AAAGGGCGATCTAGTGATGGA |
| *GAPDH*  (mouse) | Forward | AGGTCGGTGTGAACGGATTTG |
|  | Reverse | TGTAGACCATGTAGTTGAGGTCA |
| *CD32*  (mouse) | Forward | AATCCTGCCGTTCCTACTGATC |
|  | Reverse | GTGTCACCGTGTCTTCCTTGAG |
| *CD86*  (mouse) | Forward | GGCGGTGGAATACAATGTGTCC |
|  | Reverse | AGCAGGTCAAGGTGAACAGCTG |
| *SOD2*  (mouse) | Forward | GCTCCGGTTTTGGGGTATCTG |
|  | Reverse | GCGTTGATGTGAGGTTCCAG |

## Table S2. Modified neurological severity score (mNSS) assessment criteria, related to Figure S6O and S6P.

| **Motor tests** | **Score** |
| --- | --- |
| **Raise mouse by the tail** | 0-3 |
| *Flexion of forelimb* | 1 |
| *Flexion of forelimb* | 1 |
| Head moved more than 10° to vertical axis | 1 |
| **Place mouse on the floor** | 0-3 |
| Normal walk | 0 |
| Incapacity to walk straight | 1 |
| Circle toward the hemiplegia side | 2 |
| Fall down to the hemiplegia side | 3 |
| **Beam balance tests** | 0-4 |
| Maintain in stable posture | 0 |
| Hugs the beam and limb falls down from the beam | 1 |
| Hugs the beam or spins on beam | 2 |
| Attempts to balance on the beam but falls off | 3 |
| Fall off the beam | 4 |
| **Maximum points** | 10 |

## Table S3. Retrospective sensitivity power analysis for primary outcome measures, related to Figures 4, 5, 6, S6 and S7.

| **Experiment** | **Statistical test** | ***n* per group** | **Minimum detectable *f* (α = 0.05, power = 0.80)** | **Observed Cohen's *f*** | **Source** |
| --- | --- | --- | --- | --- | --- |
| TTC infarct volume (male) | One-way ANOVA | 6 | 0.48 | 3.54 | Figure.5C |
| TTC infarct volume (female) |  | 6 | 0.48 | 4.97 | Figure.S6B |
| Nissl staining |  | 6 | 0.48 | 3.68/4.50 | Figure.5E |
| Nissl staining |  | 6 | 0.48 | 6.88 | Figure.S7E |
| NeuN intensity |  | 6 | 0.48 | 2.72 | Figure.5I |
| MAP2/Cleaved Caspase-3 |  | 6 | 0.48 | 2.31 | Figure.5L |
| CD16/32⁺/Iba1⁺ cells |  | 6 | 0.48 | 5.45 | Figure.5N |
| CD206⁺/Iba1⁺ cells |  | 6 | 0.48 | 7.39 | Figure.5P |
| ELISA (IL-4, TGF-β1, TNF-α) |  | 5-6 | 0.55, 0.48 | 1.23, 2.42, 2.43 | Figure.5Q, 5R, 5S |
| MDA content |  | 3 | 1.05 | 7.05 | Figure.S6F |
| SOD activity |  | 3 | 1.05 | 9.9 | Figure.S6G |
| MRI infarct volume |  | 3 | 1.05 | 6.38 | Figure.6D |
| Brain area |  | 3 | 1.05 | 3.89 | Figure.S7B |
| Brain weight |  | 7 | 0.45 | 7.18 | Figure.S7C |
| NeuN intensity |  | 6 | 0.48 | 4.24 | Figure.6G |
| Syn1 intensity |  | 6 | 0.48 | 7.53 | Figure.6H |
| GFAP scar |  | 6 | 0.48 | 3.7 | Figure.6J |
| CD31 vessel length |  | 6 | 0.48 | 5.02 | Figure.6L |
| Balance beam | Two-way RM ANOVA | 7 | – | 1.21 | Figure.6A |
| Horizontal ladder |  | 7 | – | 1.09 | Figure.6B |
| Rotarod |  | 7 | – | 0.92 | Figure.6C |
| mNSS (male) |  | 7 | – | 2.64 | Figure.S6O |
| mNSS (female) |  | 7 | – | 2.41 | Figure.S6P |
| Brain fluorescence |  | 3 | – | 0.99 | Figure.4K |
